# Comprehensive Analysis of Histone Modifications in Hepatocellular Carcinoma Reveals Different Subtypes and Key Prognostic Models

**DOI:** 10.1155/2022/5961603

**Published:** 2022-08-01

**Authors:** Huikai Li, Han Mu, Yajie Xiao, Zhikun Zhao, Xiaoli Cui, Dongfang Wu

**Affiliations:** Department of Hepatobiliary Surgery, Tianjin Medical Cancer Institute and Hospital National Clinical Research Center for Cancer, Key Laboratory of Cancer Prevention and Therapy, Tianjin's Clinical Research Center for Cancer, Tianjin 120000, China

## Abstract

Histone modification, an important epigenetic mechanism, is related to the carcinogenesis of hepatocellular carcinoma (HCC). In three datasets, we screened 88 epigenetic-dysregulated PCGs (epi-PCGs) , which were significantly associated with HCC survival and could cluster HCC into three molecular subtypes. These subtypes were associated with prognosis, immunomodulatory alterations, and response to different treatment strategies. Based on 88 epi-PCGs in the TCGA training set, a risk prediction model composed of 4 epi-PCGs was established. The model was closely related to the clinicopathological features and showed a strong predictive ability in different clinical subgroups. In addition, the risk prediction model was an independent prognostic factor for patients with HCC. The significance of epi-PCGs in HCC is revealed by our data analysis.

## 1. Introduction

Epigenetics refers to heritable traits that are not attributable to changes in the DNA sequence [1]. *Cancer* is considered a multietiological disease and it is difficult to disentangle the contribution of a single risk factor [2]. Genetically related regulatory molecules and mechanisms have been a major concern in cancer for many years [3–5]. In recent years, there has been growing evidence supporting that epigenetic disorders, including histone modification, DNA methylation, chromatin remodeling, and the expression of noncoding RNA, play an important role in proliferation, invasion, metastasis, initiation, progression, and development in many types of human malignant tumors [6, 7]. As an important epigenetic mechanism, histone modification is a covalent posttranslational modification of histone proteins, which are comprised of methylation, phosphorylation, acetylation, ubiquitylation, and sumoylation, resulting in changes in the gene expression and cell behavior by affecting genome stability, transcription, DNA repair, and chromatin structure and function in cells [6, 8]. Abnormal gene expression and cellular behavior are the basis and main characteristics of cancer development.

Hepatocellular carcinoma originating from hepatocytes is the most common type of liver cancer and accounts for 90% of primary liver cancer. By 2030, more than 1 million people will die of this cancer [9]. A retrospective study concluded that posttranslational histone modification in HCC changes and revealed the significance of histone modification in predicting the prognosis of human cancer [10]. It was reported that low H3K9me3 expression is related to poor prognosis in patients with distal common bile duct cancer [11]. David. Seligson et al. have demonstrated in their study that lower cell levels of H3K4me2 and H3K18ac can predict adverse clinical outcomes in patients with lung and renal cell carcinoma and that lower cellular levels of H3K9me2 is also prognostic factor indicative of poorer outcome for individuals with either prostate or kidney cancers [12]. The expression of H3K9me3, H3K36me3, and H4K20me3 as epigenetic markers is linked to the survival of patients with esophageal squamous cell carcinoma [13]. Several studies have also reported histone modifications associated with HCC. An enhanced H3K4me3 level was associated with reduced overall survival of HCC [14]. The study of Kusakabe et al. showed that the H3K27me3 levels function as a prognostic marker for HCC survival [15]. Therefore, alterations in histone marks may be widespread, indicating that an integrative approach should be taken to analyze the role of histone modifications in regulating HCC.

**Figure 1:**
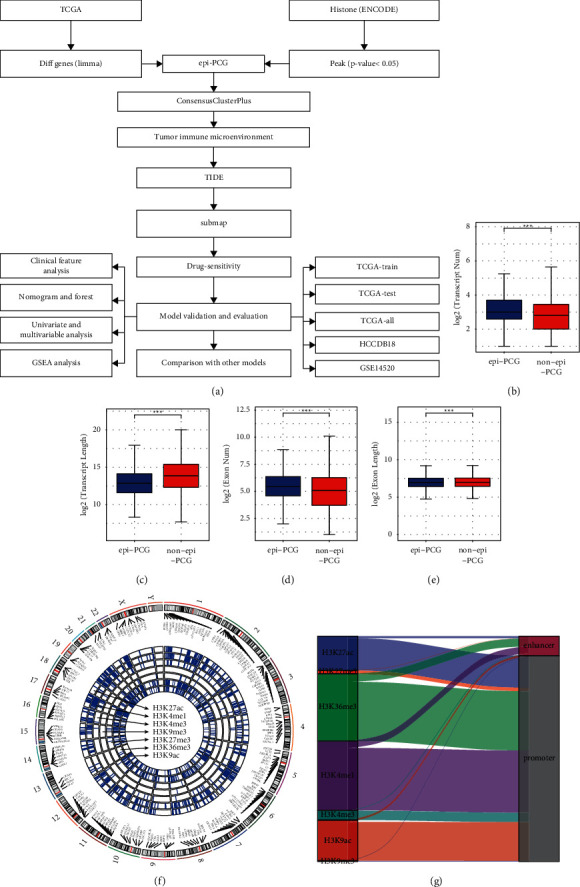
Epigenetic dysregulation of PCGs in HCC. (a): The whole workflow of the study. (b): Comparison of the number of epi-PCGs and non-epi-PCGs transcripts. (c): Wilcoxon rank sum test was used to analyze the transcript length of epi-PCGs and non-epi-PCGs. (d): Comparison of exon number between epi-PCGs and non-epi-PCGs. (e): Wilcoxon rank sum test of exon length for epi-PCGs and non-epi-PCGs. (f): Landscape of epi-PCGs with different histone modifications. (g): Distribution of apparent epi-PCGs in HCC. ^*∗*^*P* < 0.05, ^*∗∗*^*P* < 0.01, ^*∗∗∗*^*P* < 0.001.

The discovery of tumor markers is an important part of precision medicine, especially the identification of molecular subtypes, which is conducive to stratified treatment of patients. A large number of previous studies have reported the prognostic markers in a variety of liver cancers. Li et al. [16] identified 9 key genes from liver cancer based on the expression of cell death related genes, which can be used to predict the prognosis of liver cancer. Xu et al. [17] combined iron apoptosis and liver cirrhosis to construct a prognostic classifier to predict the immune prospect, chemotherapy efficacy, and immunosuppressive molecules of hepatocellular carcinoma. Huang et al. [18] integrated the epigenome and transcriptome of hepatocellular carcinoma to identify systemic enhancer aberrations and establish abnormal enhancer-related prognostic features. Xie et al. [19] identified four gene markers based on the expression of m6A-related genes and integrated multiomics data, which can be used to predict the prognosis of liver cancer. These results suggest that integrated research based on multiomics is effective in mining prognostic markers. However, these prognostic features are not used in clinical practice, especially based on the discovery of epigenetic- and transcriptomic-related markers. This means that more research is needed for clinical and experimental scientists. Integrating multiple epigenetic parameters is a powerful tool for identifying the drivers of epigenetic regulation of HCC and elucidating how epigenetic disorders lead to HCC [20]. In this study, to comprehensively analyze the role of histone modifications in HCC, we described and compared the changes of seven types of histone modifications (histone H3 trimethylated at lysine 9 (H3K9me3), histone H3 trimethylated at lysine 36 (H3K36me3), histone H3 trimethylated at lysine 27 (H3K27me3), histone H3 acetylated at lysine 9 (H3K9ac), histone H3 acetylated at lysine 27 (H3K27ac), histone H3 monomethylated at lysine 4 (H3K4me1) and histone H3 trimethylated at lysine 4 (H3K4me3)) at the promoter and enhancer elements of protein-coding genes (PCGs). Based on the epigenetic-dysregulated PCGs (epi-PCGs), HCC was divided into three subtypes, and the immune microenvironment characteristics of the three subtypes and their relationship with the response to HCC treatment were evaluated, which may provide a new insight for subtype-specific therapy. Finally, a new prognostic prediction model related to epi-PCGs was proposed with a high predictive accuracy in predicting clinical results of HCC.  Figure 1(a) shows the whole workflow of this study.

## 2. Methods

### 2.1. Transcriptome and Histone Modification Data of HCC

The transcriptome data and clinicopathological information data with HCC came from the common data set the *Cancer* Genome Atlas (TCGA)-LIHC. All HCC transcriptome data and clinicopathological information data were derived from public data sets, including the *Cancer* Genome Atlas (TCGA)-LIHC (https://portal.gdc.cancer.gov/) downloaded using TCGAbiolinks package [21]), HCCDB (http://lifeome.net/database/hccdb) and Gene Expression Omnibus (GEO (GSE14520) (http://www.ncbi.nlm.nih.gov/geo/). TCGA-LIHC contained 365 HCC samples, HCCDB18 contained 203 HCC samples, and GSE14520 contained 221 HCC samples. In addition, the seven histone modifications explored in this study were H3K9me3, H3K36me3, H3K27me3, H3K9ac, H3K27ac, H3K4me1, and H3K4me3 of human hepatoma cell lines HepG2 and normal liver tissues. Among them, H3K4me3, H3K4me1, H3K36me3, H3K9ac, and H3K27ac were associated with transcriptional activation, while H3K27me3 and H3K9me3 were associated with transcriptional inhibition. Their replicated narrowPeak data were downloaded from the Encyclopedia of DNA Elements (ENCODE) portal (https://www.encodeproject.org/).

### 2.2. Identification of Epigenetic Dysregulation Protein-Coding Genes (PCGs)

To understand the epigenetic changes in HCC, PCGs differentially expressed between HCC samples and normal liver samples were identified by Limma. Each *p* value was adjusted to FDR using the Benjamini–Hochberg (BH) method. The PCGs conforming to FDR <0.05 and | logFC | > 1 were considered to have significant statistical significance. MACS2 was used for peak detection and specific peaks of HCC were screened according to the physical location of histone-modified peaks. Peaks within *p* < 0.05 were regarded as peaks of difference. In each cell line, the upstream 2kb and downstream 0.5kb of the transcription initiation site (TSS) were defined as promoters and were recognized by ChIPseeker [22]. The enhancer data were obtained from FANTOM5 [23] and the active enhancer was determined by the H3K27ac peak. PCGs with a differential expression between normal and tumor and promoter or enhancer of them covered by at least one differential histone-modified regions (DHMR) were considered as epigenetically dysregulated PCGs.

### 2.3. Functional Annotation of Epi-PCGs

Enrichment of Gene Ontology (GO) biological process terms in epi-PCGs was assessed by computing a hypergeometric *p* value with the BH correction (FDR ≤0.05). Moreover, Kyoto Encyclopedia of Genes and Genomes (KEGG) analysis was conducted to study the tumor-related biological mechanisms of the epi-PCGs. These analyses were performed using the clusterProfiler package [24] in *R*.

### 2.4. Identification of Molecular Subtypes Related to Epi-PCGs

To establish molecular subsets related to epi-PCGs, the *R* packet ‘ConsensusClusterPlus' [25] was used to carry out consensus clustering analysis. Specifically, univariate Cox analysis was performed on epi-PCGs in TCGA-LIHC, HCCDB18, and GSE14520, respectively. Moreover, the threshold value was *p* < 0.05, which indicated that epi-PCGs were significantly linked to the prognosis of HCC. Further consensus clustering was performed for HCC samples in each dataset based on prognostic epi-PCGs in the three datasets. e. In the clustering process performed by ConsensusClusterPlus, the minimum and maximum evaluated *k* (max *k*) were set to 2 and 10, respectively, and other parameters were set to default. Moreover, the cluster number of the total HCC samples was defined by the consensus cumulative distribution function (CDF) Plot.

### 2.5. Characteristics of the Immune Microenvironment between Subgroups

To characterize the immune microenvironment of each subgroup, we analyzed the expression of chemokines, chemokine receptors, and immune checkpoints using the Kruskal–Wallis test. By uploading TCGA-LIHC gene expression data to CIBERSORT [26], immune cell scores for 22 immune cells were inferred from the gene signatures provided by CIBERSORT. In addition, single-sample gene set enrichment analysis (ssGSEA), which calculates enrichment scores to quantify the relative abundance of each immune cell in each HCC sample, was used to predict immune infiltration. Moreover, two other algorithms, microenvironment cell populations-counter (MCP-counter) and Tumor Immune Estimation Resource (TIMER), were also employed to estimate the immune cell infiltration.

### 2.6. Prediction of Response to Immuno/Chemotherapy for Each Subtype

To predict the efficacy of immunotherapy in different subtypes, we ran Tumor Immune Dysfunction and Exclusion (TIDE) algorithms and unsupervised subclass mapping method SubMap [27]. Also, the publicly available pharmacogenomics database Pharmaceutical Sensitivity Genomics in *Cancer* (GDSC) using the R-envelope tic was applied assess the chemotherapy response of each HCC sample as determined by the half-maximal inhibitory concentration (IC_50_).

### 2.7. Generation of the Risk Score Model

The samples in TCGA-LIHC were split into a training set (*n* = 182) and verification set (*n* = 183) (Table S1). In the training set, the coxph function in Survival package was used for univariate Cox analysis of prognostically related EPI-PCGs in tcGA-LIHC, HCCDB18, and GSE14520. Lasso regression analysis was performed to improve the performance parameters and decrease the false positives in variables due to overfitting. Furthermore, a two-step multivariate Cox regression analysis was used to screen for epi-PCGs significantly associated with HCC overall survival (OS). Moreover, the risk coefficients of the signature scores of the categories of genes in each sample were obtained.

### 2.8. Robustness Evaluation of Risk Scoring Models

The same risk calculation method was verified in the TCGA training dataset, TCGA validation dataset, all TCGA data sets, and two independent external datasets HCCDB18 and GSE14520. The OS was calculated using Kaplan–Meier curves, and the statistical difference was measured by the log-rank test. Moreover, “timeROC” *R* package was used to conduct a time-dependent receiver-operating characteristic (ROC) curve analysis in the training set and external and internal validation sets to evaluate the prediction accuracy of the prognostic scoring model.

### 2.9. Gene Set Variation Analysis

Gene set variation analysis (GSVA) is a method to analyze the changes of gene enrichment in a sample population in a nonparametric and unsupervised way [28]. Here, the ssGSEA method of the ‘GSVA' R package was used to calculate the scores of each sample for different functions for evaluating the association of risk scores with different functions.

### 2.10. Statistical Analysis

The *R* software (version 3.6.1) was used for statistical analysis. Clinical features and univariate and multivariate univariate and multivariate Cox survival analyses of clinical features and risk scores were used to assess the independence of the risk score model. Subgroup analysis was also carried out according to age, sex, recurrence, AJCC stage, *T* stage, N stage, *M* stage, and tumor grade stratification. Standard tests used include Student's *t*-test, Wilcoxon, Kruskal–Wallis test, one-way ANNOVA, and Fisher's exact test. All statistical tests were two-sided, two-tailed *p* < 0.05 was considered significant, which was represented by ^*∗*^, with the more ^*∗*^ showing a stronger statistical significance.

## 3. Results

### 3.1. Identification of Epigenetically Dysregulated PCGs and Their Genomic Landscape

To identify epigenetic disorders of PCGs, differences between HCC samples and normal liver samples were analyzed and a total of 2866 differential PCGs were found. We then analyzed the differential histone modification regions between HCC and normal samples and found a total of 1007 epi-PCGs and 18,435 non-epi-PCGs. The Wilcoxon rank sum test was used to analyze the number and length of exons and transcripts between epi-PCGs and non-epi-PCGs. It was found that compared with non-epi-PCGs, epi-PCGs had significantly more exons and significantly shorter transcripts (Figure 1(b), 1(c)). In terms of exons, epi-PCGs were significantly higher than non-epi-PCGs, but the length was shorter than that of non-epi-PCGs (Figure 1(d), 1I.

Next, the landscape of epigenetically dysregulated PCGs was visualized by *R* packages “Rcircos”. The circos plot showed that most of the epigenetic disorders of PCGs were usually accompanied by a variety of histone modification abnormalities and that these abnormal histone modification regions were mainly concentrated in the promoter region. The epi-PCGs of HCC were mainly regulated by H3K36me3, H3K4me1, H3K9ac, and H3K27ac of the promoter (Figure 1(f), 1(g)).

### 3.2. Biological Significance of Epigenetic Dysregulation of PCGs

**Figure 2:**
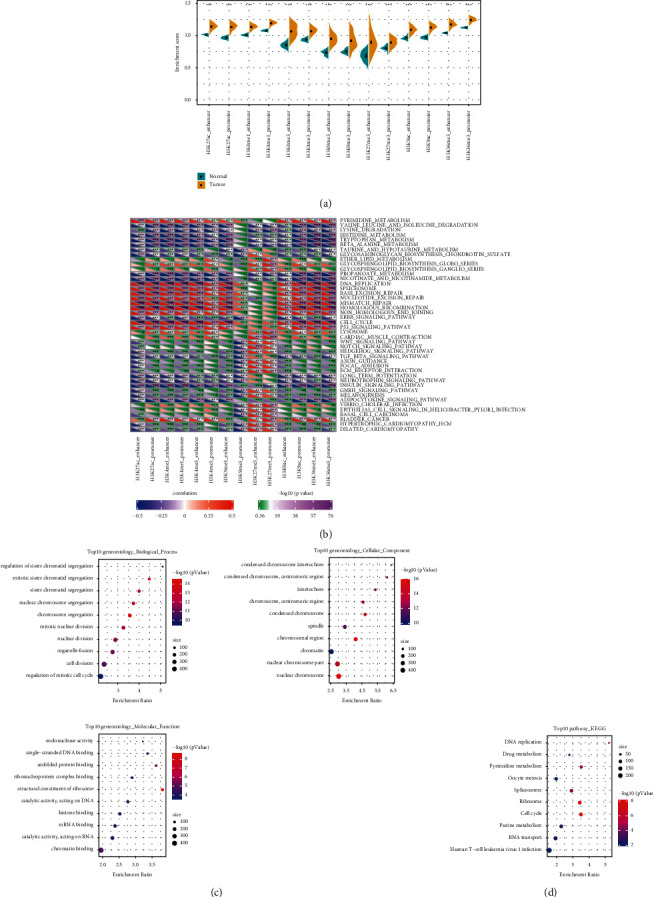
Biological significance of epigenetic dysregulation of PCGs. (a): Differences in enrichment scores of seven abnormal histone modified PCGs in promoters or enhancers between normal tissues and HCC tissues. (b): Analysis of the correlation between epi-PCGs and different HCC pathways based on the KEGG score of each KEGG sample. (c): The bubble chart shows the result of GO analysis of epi-PCGs, including top 10 pathways, cellular component, and molecular function of the biological process enrichment analysis. (d): The bubble chart shows the result of KEGG analysis in epi-PCGs. ^*∗*^*P* < 0.05, ^*∗∗*^*P* < 0.01, ^*∗∗∗*^*P* < 0.001.

To further study the function of epigenetic dysfunctional PCGs in HCC, we estimated and compared the enrichment scores of seven kinds of histone-modified PCGs in normal tissues and HCC tissues in a promoter or enhancer by ssGSEA. We observed that H3K27ac, H3K36me3, H3K4me1, and H3K9ac in the promoter and enhancer regions of epi-PCGs were significantly increased in tumor tissues compared with normal tissues. These four histone modifications positively regulate the gene expression, so gross epi-PCGs may exhibit significantly higher expression levels in tumor tissues. (Figure 2(a)). Based on the KEGG score of each sample, the relationship between epi-PCGs and different KEGG pathways was analyzed. A total of 44 pathways were associated with most histone-modified promoters and enhancers, which affected a large number of cancer pathological functions, including macromolecular biosynthesis and metabolism, cell cycle, and proliferation, etc. (Figure 2(b)). GO analysis of all epi-PCGs showed that these epi-PCGs were related to cell division and proliferation (Figure 2(c)). KEGG analysis demonstrated that epi-PCGs was significantly enriched in DNA replication, drug metabolism, cell cycle, and other biological processes (Figure 2(d)).

### 3.3. Identification and Histone Modification Analysis of Overlapping Epi-PCGs in Three Datasets

**Figure 3:**
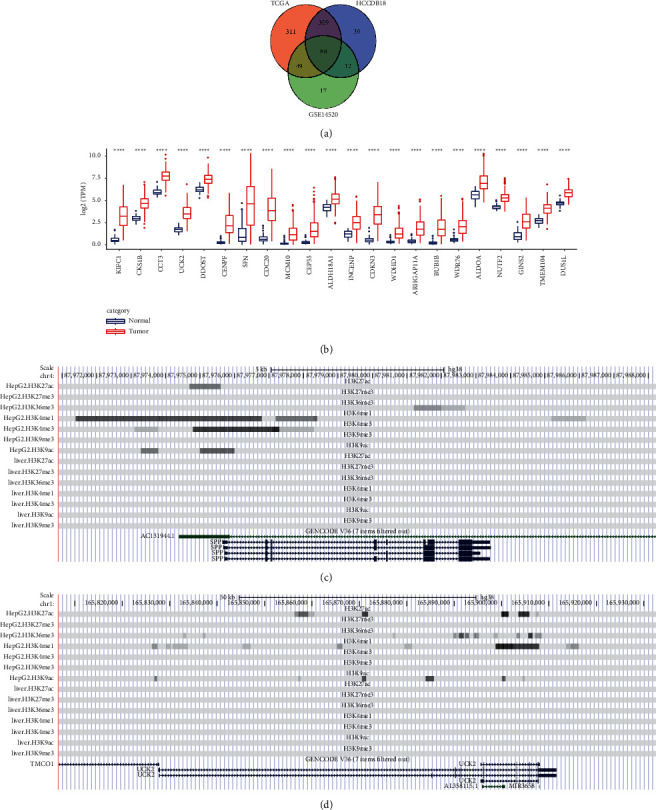
Identification and histone modification analysis of overlapping epi-PCGs in three datasets. (a): Venn diagram of epi-PCGs in TCGA-LIHC, HCCDB18 and GSE14520 that significantly related to HCC survival. (b): Wilcoxon test was used to analyze the differential expression of 22 of 88 epi-PCG between normal tissues and HCC tissues. (c): Histone modification profile of epi-PCG AC131944.1 and SPP1. (d): Histone modification profile of UCK2 and AL358115.1. ^*∗*^*P* < 0.05, ^*∗∗*^*P* < 0.01, ^*∗∗∗*^*P* < 0.001, ^*∗∗∗∗*^*P* < 0.0001.

Univariate Cox regression analysis was performed on the epi-PCGs in TCGA-LIHC, HCCDB18, and GSE14520 to obtain the epi-PCGs significantly associated with HCC survival from each data set, and the intersection was shown by a Venn diagram. The overlap contained 88 genes (Figure 3(a)). The expression of 88 epi-PCGs in normal and HCC tissues was significantly higher than that in normal tissues (Figure 3(b), Figure S1). We examined several epi-PCGs for 7 histone markers in HepG2 by UCSC genome browser. We observed that in HepG2 cells, AC131944.1 was marked with H3K4me1, H3K4me3, H3K9ac, and H3K27ac, and H3K4me1 broadly distributed both 5′ and 3′ of the TSS (Figure 3(c)). Moreover, UCK2 had obvious H3K27ac and H3K4me1 markers in HepG2 cells (Figure 3(d)).

### 3.4. Identification of Three Subtypes Based on Epi-PCGs

**Figure 4:**
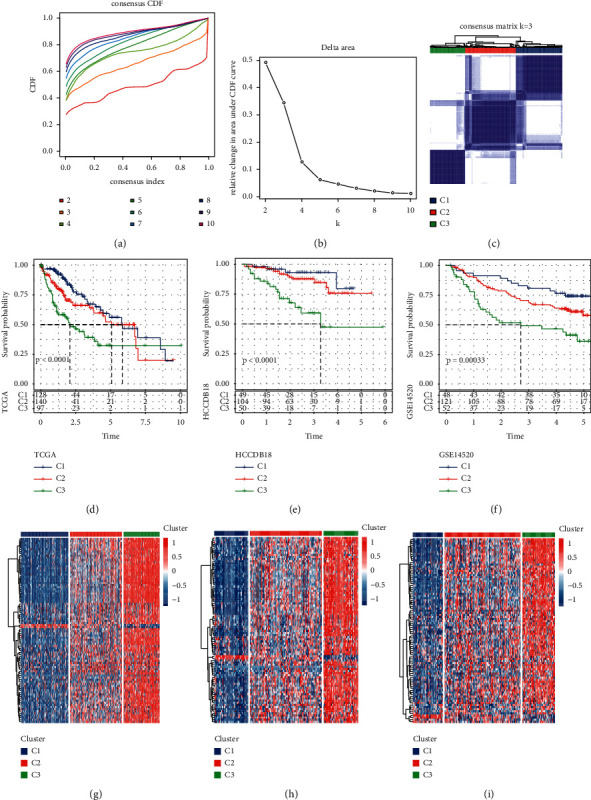
Three subtypes based on epi-PCGS were identified by consensus clustering analysis. (a): The CDF curve under different k values. (b): Relationship between the relative changes in the area under CDF curve and different k values. (c): Consensus matrix of TCGA-LIHC samples when k = 3. (d): Kaplan–Meier curves of OS among the three subgroups in TCGA-LIHC. (e): Kaplan–Meier curves among the three subtypes in the HCCDB18 database. (f): Survival curves among the three subtypes in the GSE14520 cohort. (g): Heatmap of 88 epi-PCGs expression among three subtypes in TCGA-LIHC. (h): Heatmap showed the expression of 88 epi-PCGs among three subtypes in the HCCDB18 cohort. (i): Expression heatmap of 88 epi-PCGs in three subtypes of the GSE14520 cohort.

To explore the histone modification pattern of HCC, unsupervised consensus clustering of 88 epi-PCGs shared by three data sets was conducted. According to the cumulative distribution function (CDF) and the area under the CDF curve, the optimal *k* value was 3, indicating that HCC could be divided into three subtypes (Figure 4(a) and 4(b)). Consensus matrix of HCC samples in three data sets showed that the three subgroups were arranged into three well-defined regions with internal uniqueness (Figure 4(c)). The relative changes of CDF and area under CDF curve under different *k* values of the two external data sets HCCDB18 and GSE14520 and consensus matrix when *k* = 3 are shown in Figure S2. Survival analysis revealed significant OS differences among the three subtypes in each dataset. The survival rate from high to low was *C*1 > C2 > C3 (Figures 4(d)–4(f)). Moreover, we also analyzed the expression of epi-PCG-defined HCC subtypes. The results were displayed by a heatmap. There was a significant lack of highly expressed epi-PCGs in C1, and quite a number of EPI-PCGs were significantly overexpressed in C3 (Figures 4(g)–4(i)).

We compared the expression differences of epi-PCGs between different subtypes. 288 epi-PCGs were differentially expressed between C1 and C2, including 277 downregulated epi-PCGs in C1 that related to cell cycle and 11 upregulated epi-PCGs that enriched in GO terms and KEGG pathways related to immune (Figure S3). 876 epi-PCGs were differentially expressed between C1 and C3, including 844 downregulated epi-PCGs in C1 that enriched in GO terms and KEGG pathways related to cell cycle and metabolism and 32 upregulated epi-PCGs that related to metabolism (Figure S4). 463 epi-PCGs were differentially expressed between C2 and C3, including 429 downregulated epi-PCGs in C2 that enriched in GO terms and KEGG pathways related to cell proliferation and 34 upregulated epi-PCGs that related to catabolic and metabolic process (Figure S5).

### 3.5. Characterization of the Immune Microenvironment in Three Histone-Modified Isoforms

**Figure 5:**
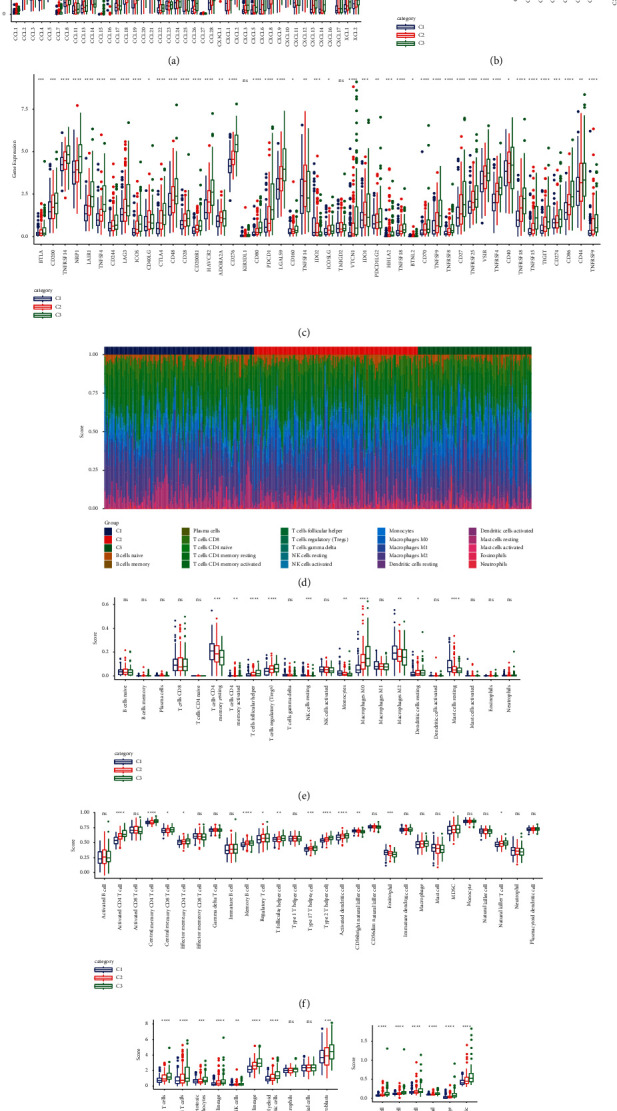
Analysis of immune-related indexes among three subtypes. (a): The relative expression of chemokines in each subtype. (b): Relative expression of chemokine receptors among three subtypes. (c): Kruskal–Wallis test was used to compare the expression of immune checkpoints among subgroups. (d): The score of 22 infiltrating immune cells in each sample of each subtype of TCGA-LIHC. (e): The score of immune cells in HCC subtype TME based on the CIBERSORT algorithm. (f): The box diagram showed the enrichment fraction of 28 immune cell clusters in each subtype. (g): The infiltration of immune cell predicted by MCP-counter in three subgroups. (h): Immune cell infiltration among three subgroups was investigated via TIMER. ^*∗*^*P* < 0.05, ^*∗∗*^*P* < 0.01, ^*∗∗∗*^*P* < 0.001, ^*∗∗∗∗*^*P* < 0.0001.

In TME, chemokines are produced by tumor cells, mesenchymal stem cells (MSC), endothelial cells, neutrophils, cancer-associated fibroblasts, and bone marrow cells, providing a very rich “soil” to facilitate the recruitment of immune cells into the TME [29]. Tumor cell-associated immune checkpoint molecules, whose primary function is thought to be mediating immune evasion, also play an important role in maintaining many malignant behaviors, including self-renewal, anti-apoptosis, angiogenesis, epithelial-mesenchymal transformation, and metastasis [30]. To confirm the TME-related molecules characteristics of each subtype, the expression of chemokines, chemokine receptors, and immune checkpoint genes were detected. The expression of 41 chemokines in TCGA-LIHC in three subtypes was analyzed. It was found that 30 chemokines were significantly differentially expressed in the three subtypes (Figure 5(a)). Among the 18 chemokine receptors detected, 16 showed significant differences in expression among the three subtypes except KIR3DL1 and TMIGD2 (Figure 5(b)). In addition, expression analysis of 47 immune checkpoint gene [31] demonstrated that almost all of the immune checkpoints had significantly different levels of expression in the three subtypes (Figure 5(c)). The expression of most of these chemokines, chemokine receptors, and immune checkpoints were significantly higher in C3 than in C2, and higher in C2 than in C1.

The imbalance of immune-related molecules among subtypes encouraged the further study of the characteristics of immune infiltration in TME. According to the CIBERSORT algorithm, all cell subsets were distinguished and the scores of 22 kinds of immune cells were calculated, and it was found that significant intergroup differences was in 10 types of immune cell populations among the three subtypes. C1 showed significantly higher activated memory CD4 T cells, resting NK cells, monocytes, M2 macrophages, and resting mast cell immune scores than C2 and C3. In contrast, C3 showed significantly higher helper follicular T cells and M0 macrophages and resting dendritic cells immune scores than C1 and C2 (Figure 5(d), 5(e)). Moreover, ssGSEA was used to estimate the infiltration of 28 immune cells and to display intersubtype differences in immune cells by boxplots. Half of the immune cell clusters showed significant differences in the scores of the three subtypes. The scores of 11 kinds of immune cells in C2 and C3 were significantly higher than those in the C1 subtype (Figure 5(f)). In the results of MCP-counter analysis, the scores of T cells, CD8 T cells, cytotoxic lymphocytes, B cells, NK cells, myeloid dendritic cells, and fibroblasts in C2 and C3 were significantly upregulated compared with those of C1 (Figure 5(g)). We also observed high infiltration of B cell, CD4 T cell, CD8 T cell, neutrophil, macrophage, and dendritic in C3 in the results of TIMER evaluation (Figure 5(h)). These results indicated the heterogeneity of immune infiltration among the three subtypes.

### 3.6. Evaluation of the Treatment Response of HCC Subtypes

**Figure 6:**
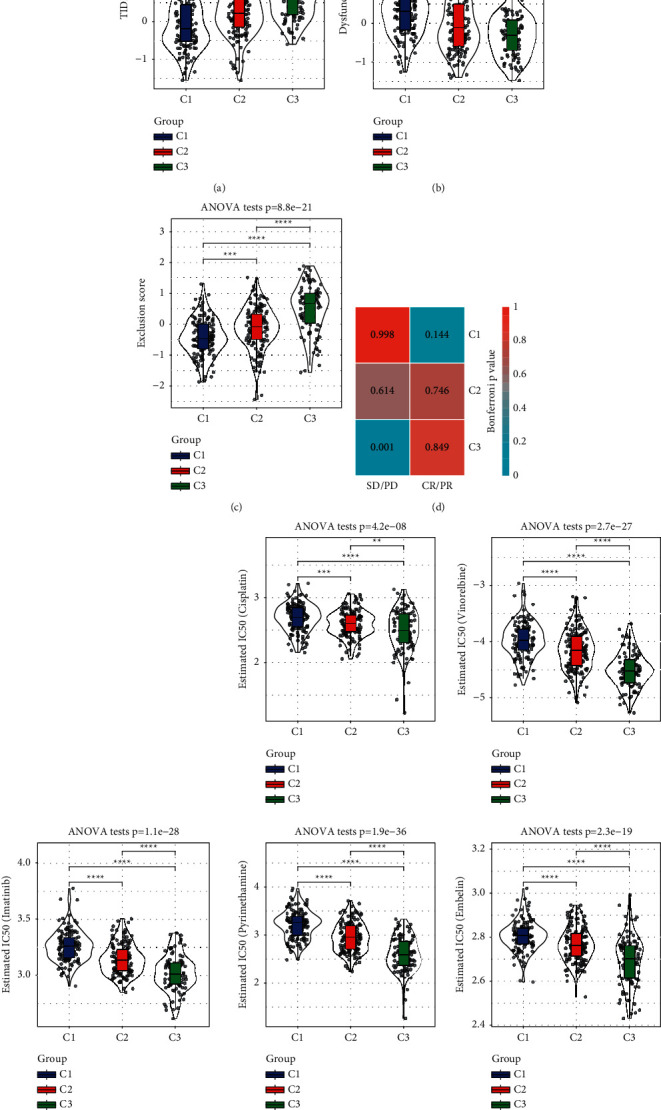
Evaluation of therapeutic response of HCC subtypes in TCGA Potential response to immunotherapy in samples from the different subtypes was assessed by TIDE score (a), T-cell dysfunction score (b), and T-cell rejection score (c). (d): Submap analysis data revealed that samples of C3 subtype were resistant to immunotherapy.SD: stable disease; PD: progressive disease; CR: complete response; PR: partial response. (e): The violin diagrams of predicted IC50 values of cisplatin cisplatin, vinorelbine, imatinib, pyrimethamine, and embelin based on GDSC database drugs in three subtypes of TCGA-LIHC dataset. ^*∗*^*P* < 0.05, ^*∗∗*^*P* < 0.01, ^*∗∗∗*^*P* < 0.001, ^*∗∗∗∗*^*P* < 0.0001.

The difference in TME among the three subtypes prompted us to study the response of each subgroup to immunotherapy. A potential response to immunotherapy in samples from the different subtypes was modeled on TIDE instructions, and T-cell dysfunction and rejection were used to predict the performance of ICBs in the three subtypes. There were significant differences in the TIDE score and T-cell dysfunction score and exclusion score among the three subgroups. Among the three subtypes, C1 had the lowest TIDE score and T-cell exclusion score and the highest T-cell dysfunction score. Different from C1, C3 had the highest TIDE score and T-cell exclusion score and the lowest T-cell dysfunction score. All three scores of C2 were in the middle (Figure 6(a)-6(c)). Submap analysis data revealed that samples of C3 subtype were resistant to immunotherapy (Figure 6(d)). The response of the sample to antineoplastic drugs was evaluated in three subtypes, such as cisplatin, vinorelbine, imatinib, pyrimethamine, and embelin. The sensitivity of the three subtypes to several drugs was different. Among the three subtypes, C3 was more sensitive to cisplatin, vinorelbine, imatinib, pyrimethamine, or embelin. C1 was not sensitive to the above five antineoplastic drugs (Figure 6(e)).

### 3.7. Construction of a Prognostic Prediction Model Based on Four Epi-PCGs

**Figure 7:**
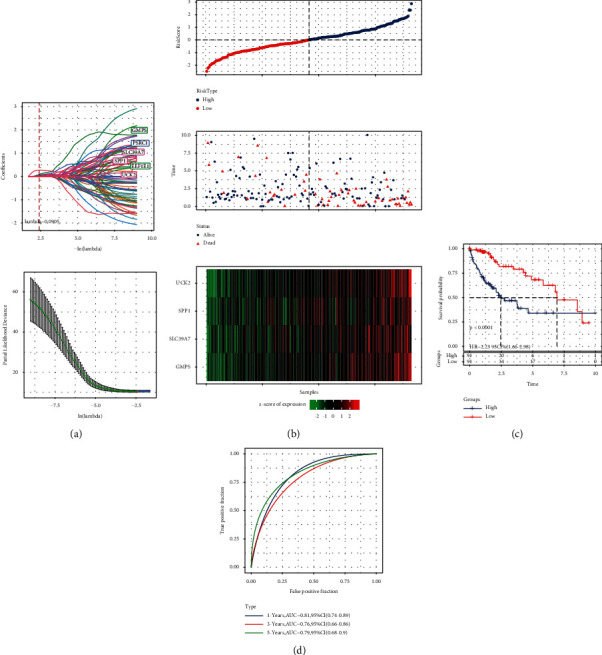
The prognostic prediction model was constructed based on the analysis of epi-PCGs in the training set. (a): LASSO Cox regression analysis was used to identify the prognosis of epi-PCGs, the best *λ* = 0.0905. (b): The corresponding risk score, life status, and expression level of 4 genes in the training set. (c): Kaplan–Meier survival curves of training set samples. (d): Time-dependent ROC curve of prognostic prediction model.

To develop a specific prognostic tool for predicting HCC, we established a risk model based on the expression data of epi-PCGs. During the training, 88 epi-PCGs obtained by Figure 3(a) were included in univariate Cox regression analysis and 71 epi-PCGs closely related to the prognosis of HCC patients were identified. Six genes significantly associated with the prognosis of HCC were screened by the Lasso Cox analysis based on the optimal *λ* value (*λ* = 0.0905) (Figure 7(a)). The stepAIC based on Akaike information criterion (AIC) further eliminated two epi-PCGs, and the remaining four epi-PCGs were used to construct risk score signature: Risk score = 0.361 × UCK2 + 0.064 × SPP1 + 0.365 × GMPS + 0.321 × SLC39A7. The risk score, life status, and expression level of four genes in the training set showed that the number of HCC-specific death in high-risk patients was higher than that in low-risk patients, and all four genes were high-risk genes, and the expression increased with the increase of risk score (Figure 7(b)). Similarly, survival analysis of the training set showed significantly higher mortality rates among high-risk patients than among low-risk patients (Figure 7(c)). The ROC curve showed that the AUC of 1-year, 3-year, and 5-year survival of patients in the training group were 0.81, 0.76, and 0.79, respectively (Figure 7(d)).

### 3.8. Internal and External Verification of the Risk Model

**Figure 8:**
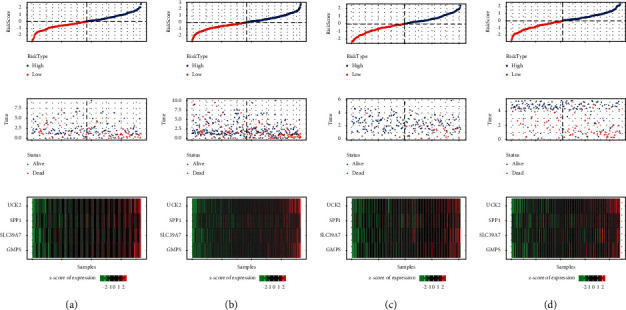
The corresponding risk score of the samples in the four verification cohorts, the relationship between the survival time and the expression level of 4 genes, including (a): TCGA internal validation set, (b): the whole TCGA-LIHC data set, (c): external validation cohort HCCDB18, and (d): GSE14520.

**Figure 9:**
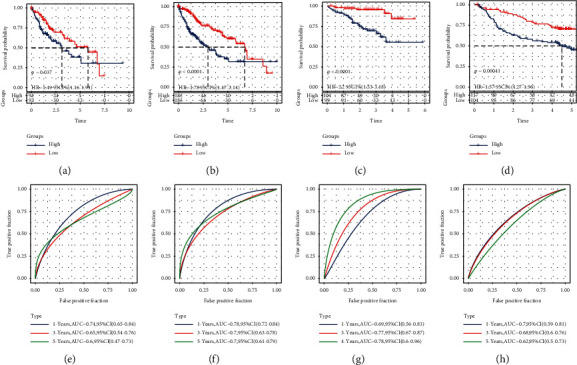
Prognostic validation of the risk prediction model. (a-d): K–M curves evaluating the prognostic prediction model between low- and high-risk groups in the internal validation set, the entire TCGA-LIHC cohort, and the external validation independent cohort HCCDB18 and GSE14520 cohorts. (e-f): ROC curves of prognostic prediction model in the internal validation set, the entire TCGA-LIHC cohort, the HCCDB18 cohort, and GSE14520 cohort.

The prognostic prediction model performance to predict OS was validated in the internal validation set, the entire TCGA-LIHC cohort, and two external cohorts. In each cohort, each sample was assigned a risk score and arranged from low to high. In both the TCGA-LIHC validation set and the entire TCGA-LIHC cohort, increased risk scores were associated with increased mortality and upregulated gene expression in risk models (Figure 8(a)-8(b)). In the two external validation cohorts HCCDB18 and GSE14520, the death rate of samples in the high-risk group was much higher than that in the low-risk group, and the expression of four genes in the risk model was also significantly increased in the high-risk group compared with the low-risk group (Figures 8(c)–8(d)). The relationship between the risk score and survival time and the expression of four epi-PCGs in prognostic prediction model in high and low-risk samples were summarized, and the outcome trend was consistent with the training set (Figure 8). The prognostic significance of the prediction model was explored by collating the transcriptome data and survival information of each cohort. In all validation cohorts, the 5-year survival rate in the low-risk group was significantly higher than that in the low-risk group (Figure 9(a)–9(d)). The 1-year AUCs for samples in the internal validation set, the entire TCGA-LIHC cohort and the external validation sets HCCDB18 and GSE14520 were 0.74, 0.78, 0.69, and 0.7, respectively. The 3-year AUC value for samples in the four cohorts was 0.65, 0.78, 0.77, and 0.68, respectively. Moreover, the 5-year AUC for samples in the four cohorts was 0.6, 0.7, 0.78, and 0.62, respectively (Figures 9(e)–9(h)).

### 3.9. Risk Model Had an Independent Prognostic Value for HCC

**Figure 10:**
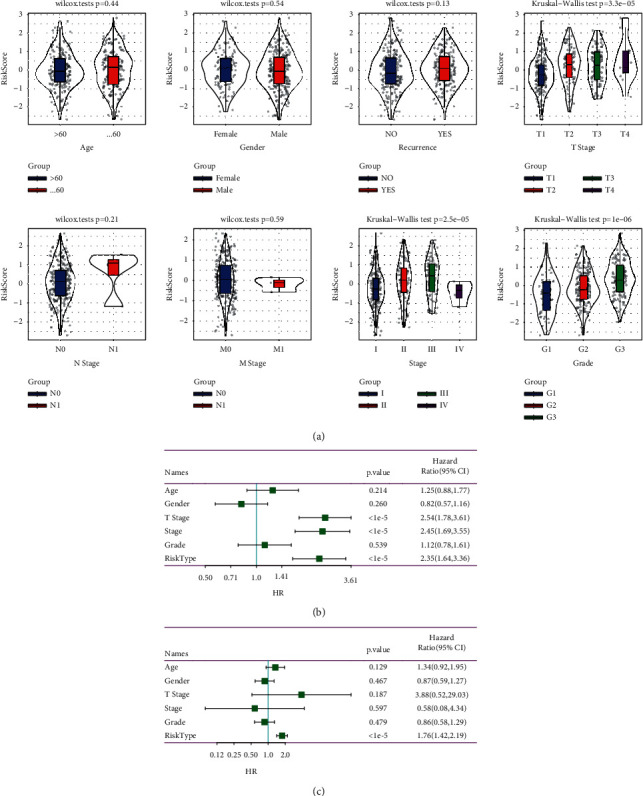
Independent prognostic performance assessment of risk models. (a): Relationship between risk scores and clinical features, including risk score and age, risk score and gender，risk score and recurrence，risk score and T stage, risk score and N stage, risk score and M stage, risk score and AJCC stage, and risk score and clinical grade. (b): Univariate analysis and based on the risk score and clinical manifestation. (c) Multivariate analysis based on the risk score and clinical features. Multivariate Cox regression analyses evaluated the prognostic independence of the prognostic prediction model regarding OS in the entire TCGA-LIHC cohort.

To determine the relationship between prognostic prediction model and clinical features, the risk score distribution of clinicopathological information including age, sex, recurrence, *T* stage, N stage, *M* stage, AJCC stage, and clinical grade in the whole TCGA-LIHC data set was analyzed. These results showed that patients with a high-risk score tended to include those who had more advanced *T* stage, AJCC stage, and clinical grade. There was no statistical correlation between the established prognostic prediction model and age, sex, recurrence, N stage, and *M* stage (Figure 10(a)). Univariate and multivariate Cox regression analyses were performed to determine the independence of these clinicopathological characteristics and risk score in predicting HCC prognosis. Only the risk score was proved as an independent prognostic factor for HCC (Figure 10(b), 10(c)).

### 3.10. Predictive Role of Survival by Risk Models in a Variety of Clinical Features

**Figure 11:**
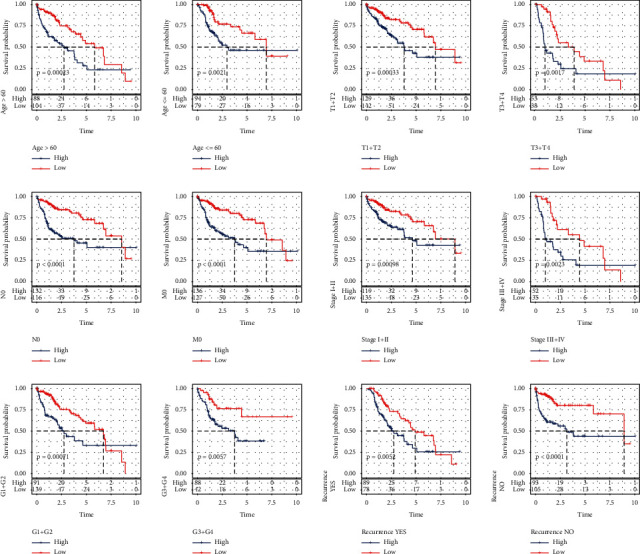
Kaplan–Meier analysis of OS for HCC samples stratified by age (age≤ 60/> 60), T stage (T1-T2/T3-T4), N stage (N0), M stage (M0), AJCC stage (I-II/III-IV), clinical grade(G1-G2/G-G4), and recurrence or not.

Finally, the whole TCGA-LIHC sample was stratified according to clinical parameters, including age (age ≤60/> 60), *T* stage (T1-T2/T3-T4), N stage (N0), *M* stage (M0), AJCC stage (I-II/III-IV), clinical grade (G1-G2/G-G4), and recurrence or nonrecurrence. The results showed that the 5-year OS of high-risk patients with age, sex, *T* stage, AJCC stage, early N stage, early *M* stage, and clinal grade stage was significantly shorter than that of low-risk patients (Figure 11).

## 4. Discussion

HCC is one of the leading causes of cancer death worldwide. HCC was closely related to the change of histone modification [32]. It is still an important task to find the key genes in the histone modification related to HCC. In this study, we aimed to reveal different molecular subtypes of HCC by identifying key genes related to histone modification and to explore the key histone-modified gene signature affecting the prognosis of HCC.

In this study, we identified 1007 epi-PCGs in HCC samples that were different from normal samples. The landscape of epigenetically dysregulated PCGs revealed different epigenetic patterns, which were mainly regulated by promoters H3K36me3, H3K4me1, and H3K9ac, and they also regulate key biological functions in the development of HCC, such as metabolism, cell cycle, and proliferation. In some past studies, several subtypes of HCC have been identified based on transcriptomic abnormalities and genetic alterations that are closely related to risk factors, pathological features, and prognosis [33]. The three HCC subclasses identified in this study were defined based on 88 of 1007 epigenetic-dysregulated PCGs. Among the three subtypes, the OS of C1 was the best, the expression of chemokine, its receptor, and immune checkpoint was the lowest in C1, and activated memory CD4 T cells, resting NK cells, M2 macrophages, and resting mast cells were more active. Thus, C1 may be more inclined to inhibit tumorigenesis [34]. The prognosis of C3 was the worst, the expression of most immune-related molecular indicators was the highest, and the immune scores of helper follicular T cells and M0 macrophages and resting dendritic cells were higher. These are the favorable characteristics to maintain the malignant progression of the tumor [35]. It is speculated that high levels of chemokines and their receptors and immune checkpoints block their anti-tumor immune response, resulting in poor prognosis of this subtype [36]. Although C3 had the highest expression of immune checkpoint, it should be noted that samples of C3 subtype were resistant to immunotherapy.

Finally, a prognostic prediction model based on four epi-PCGs (UCK2, SPP1, GMPS, and SLC39A7) was established through a step-by-step bioinformatics analysis of overlapping epi-PCGs in three subtypes. We analyzed the correlation between the methylation level of these four gene promoter regions and gene expression. First, we can observe that the methylation of SLC39A7, SPP1, and UCK2 promoter regions was significantly negatively correlated with gene expression, suggesting that the expression of these genes may also be affected by methylation (Figure S6(a)-S6(d)). Although the expression difference in the chemotherapy response group was not observed (Figure S6(e)), it can be seen that SPP1 had significantly high expression in the radiotherapy response group (Figure S6(f)). Some of these epi-PCGs have been identified as risk factors for many cancers. UCK2 is a carcinogenic driving gene in lung cancer. It has high diagnostic accuracy and is associated with poor clinicopathological features, including higher *T* and N stages, as well as a higher probability of early recurrence [37]. Recent studies have shown that UCK2 was a cancer-promoting factor in HCC and was associated with adverse clinical outcomes of HCC [38, 39]. The role of SPP1 has been validated in several types of cancers, including in esophageal cancer [40], ovarian cancer [41], lung cancer [42], gastric cancer [43], and breast cancer [44]. Its cancer-promoting effect and mechanism in HCC have also been widely studied [45, 46]. A recent report showed that the inhibition of GMPS could block glutamine metabolism and the growth of prostate cancer [47]. Kerstin Holzer et al. revealed that GMPS was a target for p53 inhibition in hepatocellular carcinoma by proteomic analysis [48]. Moreover, GMPS was also reported to be a specific blood biomarker for the diagnosis of human HCC [49]. In addition, the role of SLC39A7 in a variety of malignant tumors such as gastric cancer [50], cervical cancer [51], colorectal cancer [52], and prostate cancer [53] has also been studied, but these studies on malignant tumors do not include liver cancer, but they also increase the credibility of our results.

In our study, the abovementioned four epi-PCGs were combined into a single panel, which could relatively accurately distinguish the OS of patients with HCC of different risks. Stratified survival analysis showed that the risk model was closely related to clinicopathological features. Therefore, our risk prediction model had great potential in guiding personalized therapy for HCC patients.

## 5. Conclusions

In summary, our study analyzed different epigenetic modifications of epi-PCGs and revealed three different molecular subsets of HCC by identifying key genes related to histone modification, which were related to prognosis, immunomodulatory changes, and responses to different treatment strategies. Finally, a risk prediction model based on four epi-PCGs was developed, which was an independent prognostic factor of HCC and performed well in individual risk stratification and survival prediction of HCC patients.

## Data Availability

The data used to support the results can be found in the TCGA (https://portal.gdc.cancer.gov), HCCDB (http://lifeome.net/database/hccdb), GEO (GSE14520, http://www.ncbi.nlm.nih.gov/geo/), and ENCODE (https://www.encodeproject.org/). The codes used and/or analyzed during the current study are available from the corresponding author on reasonable request.

## Supplementary Materials

Supplementary Materials Supplementary Materials. Figure S1. Wilcoxon test was used to analyze the differential expression of 66 of 88 epi-PCG between normal tissues and HCC tissues. Figure S2. The relative changes of CDF and area under CDF curve under different *k* values of the two external data sets HCCDB18(A) and GSE14520(B) and consensus matrix when *k* = 3. Figure S3. GO and KEGG analysis of differences epi-PCGs between C1 and C2. A : Significantly downregulated epi-PCGs enriched GO terms and KEGG pathway in C1 compared with C2. B : Significantly upregulated epi-PCGs enriched GO terms and KEGG pathway in C1 compared with C2. Figure S4. GO and KEGG analysis of differences epi-PCGs between C1 and C3. A : Significantly downregulated epi-PCGs enriched GO terms and KEGG pathway in C1 compared with C3. B : Significantly up-regulated epi-PCGs enriched GO terms and KEGG pathway in C1 compared with C3. Figure S5. GO and KEGG analysis of differences epi-PCGs between C2 and C3. A : Significantly downregulated epi-PCGs enriched GO terms and KEGG pathway in C2 compared with C3. B : Significantly upregulated epi-PCGs enriched GO terms and KEGG pathway in C2 compared with C3. Figure S6. A : Correlation between methylation of GMPS promoter region and gene expression. B : Correlation between methylation of SLC39A7promoter region and gene expression. C : Correlation between methylation of SPP1 promoter region and gene expression. D : Correlation between methylation of UCK2 promoter region and gene expression. E : Distribution difference of four gene expression in chemotherapy response group. F : Distribution difference of four gene expression in radiotherapy response group. Table S1. Clinicopathological features between the training set and the validation set.

## References

[1] Brien G. L., Valerio D. G., Armstrong S. A. (2016). Exploiting the epigenome to control cancer-promoting gene-expression programs. *Cancer Cell*.

[2] Liu H., Shi W., Jin Z. (2021). Global, regional, and national mortality trends of female breast cancer by risk factor, 1990-2017. *BMC Cancer*.

[3] Shi W., Hu D., Lin S., Zhuo R. (2020). Five-mRNA signature for the prognosis of breast cancer based on the ceRNA network. *BioMed Research International*.

[4] Hu D. J., Shi W. J., Yu M., Zhang L. (2019). High WDR34 mRNA expression as a potential prognostic biomarker in patients with breast cancer as determined by integrated bioinformatics analysis. *Oncology Letters*.

[5] Chen Z., Wang M., De Wilde R. L. (2021). A machine learning model to predict the triple negative breast cancer immune subtype. *Frontiers in Immunology*.

[6] Ellis L., Atadja P. W., Johnstone R. W. (2009). Epigenetics in cancer: targeting chromatin modifications. *Molecular Cancer Therapeutics*.

[7] Wahid B., Ali A., Rafique S., Idrees M. (2017). New insights into the epigenetics of hepatocellular carcinoma. *BioMed Research International*.

[8] Rajan P. K., Udoh U. A., Sanabria J. D. (2020). The role of histone acetylation-/methylation-mediated apoptotic gene regulation in hepatocellular carcinoma. *International Journal of Molecular Sciences*.

[9] Villanueva A. (2019). Hepatocellular carcinoma. *New England Journal of Medicine*.

[10] Zhang H., Shang Y.-P., Chen H.-y., Li J. (2017). Histone deacetylases function as novel potential therapeutic targets for cancer. *Hepatology Research*.

[11] Kim H. G., Sung J.-Y., Na K., Kim S.-W. (2020). Low H3K9me3 expression is associated with poor prognosis in patients with distal common bile duct cancer. *In Vivo*.

[12] Seligson D. B., Horvath S., McBrian M. A. (2009). Global levels of histone modifications predict prognosis in different cancers. *American Journal Of Pathology*.

[13] Zhou M., Li Y., Lin S. (2019). H3K9me3, H3K36me3, and H4K20me3 expression correlates with patient outcome in esophageal squamous cell carcinoma as epigenetic markers. *Digestive Diseases and Sciences*.

[14] He C., Xu J., Zhang J. (2012). High expression of trimethylated histone H3 lysine 4 is associated with poor prognosis in hepatocellular carcinoma. *Human Pathology*.

[15] Kusakabe Y., Chiba T., Oshima M. (2021). EZH1/2 inhibition augments the anti-tumor effects of sorafenib in hepatocellular carcinoma. *Scientific Reports*.

[16] Li H., Li T., Zhang X. (2022). Identification of a pyroptosis-related prognostic signature combined with experiments in hepatocellular carcinoma. *Frontiers in Molecular Biosciences*.

[17] Xu L., Zheng Q., Liu W. (2022). Combination of ferroptosis and pyroptosis to construct a prognostic classifier and predict immune landscape, chemotherapeutic efficacy and immunosuppressive molecules in hepatocellular carcinoma. *BMC Cancer*.

[18] Huang P., Zhang B., Zhao J., Li M. D. (2022). Integrating the epigenome and transcriptome of hepatocellular carcinoma to identify systematic enhancer aberrations and establish an aberrant enhancer-related prognostic signature. *Frontiers in Cell and Developmental Biology*.

[19] Xie S., Wang Y., Huang J., Li G. (2022). A novel m6A‐related prognostic signature for predicting the overall survival of hepatocellular carcinoma patients. *IET Systems Biology*.

[20] Hlady R. A., Sathyanarayan A., Thompson J. J. (2019). Integrating the epigenome to identify drivers of hepatocellular carcinoma. *Hepatology*.

[21] Colaprico A., Silva T. C., Olsen C. (2016). TCGAbiolinks: an R/Bioconductor package for integrative analysis of TCGA data. *Nucleic Acids Research*.

[22] Yu G., Wang L.-G., He Q.-Y. (2015). ChIPseeker: an R/Bioconductor package for ChIP peak annotation, comparison and visualization. *Bioinformatics*.

[23] Abugessaisa I., Noguchi S., Carninci P., Kasukawa T. (2017). The FANTOM5 computation ecosystem: genomic information hub for promoters and active enhancers. *Methods in Molecular Biology*.

[24] Yu G., Wang L.-G., Han Y., He Q.-Y. (2012). clusterProfiler: an R package for comparing biological themes among gene clusters. *OMICS: A Journal of Integrative Biology*.

[25] Wilkerson M. D., Hayes D. N. (2010). ConsensusClusterPlus: a class discovery tool with confidence assessments and item tracking. *Bioinformatics*.

[26] Newman A. M., Liu C. L., Green M. R. (2015). Robust enumeration of cell subsets from tissue expression profiles. *Nature Methods*.

[27] Hoshida Y., Brunet J.-P., Tamayo P., Golub T. R., Mesirov J. P. (2007). Subclass mapping: identifying common subtypes in independent disease data sets. *PLoS One*.

[28] Hänzelmann S., Castelo R., Guinney J. (2013). GSVA: gene set variation analysis for microarray and RNA-seq data. *BMC Bioinformatics*.

[29] Vilgelm A. E., Richmond A. (2019). Chemokines modulate immune surveillance in tumorigenesis, metastasis, and response to immunotherapy. *Frontiers in Immunology*.

[30] Zhang Y., Zheng J. (2020). Functions of immune checkpoint molecules beyond immune evasion. *Advances in Experimental Medicine and Biology*.

[31] Danilova L., Ho W. J., Zhu Q. (2019). Programmed cell death ligand-1 (PD-L1) and CD8 expression profiling identify an immunologic subtype of pancreatic ductal adenocarcinomas with favorable survival. *Cancer Immunology Research*.

[32] Galicia-Moreno M., Silva-Gomez J. A., Lucano-Landeros S., Santos A., Monroy-Ramirez H. C., Armendariz-Borunda J. (2021). Liver cancer: therapeutic challenges and the importance of experimental models. *Chinese Journal of Gastroenterology and Hepatology*.

[33] Rebouissou S., Nault J.-C. (2020). Advances in molecular classification and precision oncology in hepatocellular carcinoma. *Journal of Hepatology*.

[34] Bule P., Aguiar S. I., Aires-Da-Silva F., Dias J. N. R. (2021). Chemokine-directed tumor microenvironment modulation in cancer immunotherapy. *International Journal of Molecular Sciences*.

[35] Singh S., Sadanandam A., Singh R. K. (2007). Chemokines in tumor angiogenesis and metastasis. *Cancer metastasis reviews*.

[36] Zhou P., Lu Y., Xun Y. (2021). Ubiquitin modification patterns of clear cell renal cell carcinoma and the ubiquitin score to aid immunotherapy and targeted therapy. *Frontiers in Cell and Developmental Biology*.

[37] Wu Y., Jamal M., Xie T. (2019). Uridine‐cytidine kinase 2 (UCK2): a potential diagnostic and prognostic biomarker for lung cancer. *Cancer Science*.

[38] Yu S., Li X., Guo X., Zhang H., Qin R., Wang M. (2019). UCK2upregulation might serve as an indicator of unfavorable prognosis of hepatocellular carcinoma. *IUBMB Life*.

[39] Cai J., Sun X., Guo H. (2020). Non-metabolic role of UCK2 links EGFR-AKT pathway activation to metastasis enhancement in hepatocellular carcinoma. *Oncogenesis*.

[40] Li M., Wang K., Pang Y. (2020). Secreted phosphoprotein 1 (SPP1) and fibronectin 1 (FN1) are associated with progression and prognosis of esophageal cancer as identified by integrated expression profiles analysis. *Medical Science Monitor: International Medical Journal of Experimental and Clinical Research*.

[41] Zeng B., Zhou m., Wu H., Xiong Z. (2018). SPP1 promotes ovarian cancer progression via Integrin &beta;1/FAK/Akt signaling pathway. *OncoTargets and Therapy*.

[42] Tang H., Chen J., Han X., Feng Y., Wang F. (2021). Upregulation of SPP1 is a marker for poor lung cancer prognosis and contributes to cancer progression and cisplatin resistance. *Frontiers in Cell and Developmental Biology*.

[43] Wang Y., Zheng K., Chen X., Chen R., Zou Y. (2021). Bioinformatics analysis identifies COL1A1, THBS2 and SPP1 as potential predictors of patient prognosis and immunotherapy response in gastric cancer. *Bioscience Reports*.

[44] Göthlin Eremo A., Lagergren K., Othman L., Montgomery S., Andersson G., Tina E. (2020). Evaluation of SPP1/osteopontin expression as predictor of recurrence in tamoxifen treated breast cancer. *Scientific Reports*.

[45] Zhu Y., Yang J., Xu D. (2019). Disruption of tumour-associated macrophage trafficking by the osteopontin-induced colony-stimulating factor-1 signalling sensitises hepatocellular carcinoma to anti-PD-L1 blockade. *Gut*.

[46] Nardo A. D., Grün N. G., Zeyda M. (2020). Impact of osteopontin on the development of non‐alcoholic liver disease and related hepatocellular carcinoma. *Liver International*.

[47] Wang Q., Guan Y. F., Hancock S. E. (2021). Inhibition of guanosine monophosphate synthetase (GMPS) blocks glutamine metabolism and prostate cancer growth. *The Journal of Pathology*.

[48] Holzer K., Drucker E., Roessler S. (2017). Proteomic analysis reveals GMP synthetase as p53 repression target in liver cancer. *American Journal Of Pathology*.

[49] Yin L., He N., Chen C., Zhang N., Lin Y., Xia Q. (2019). Identification of novel blood-based HCC-specific diagnostic biomarkers for human hepatocellular carcinoma. *Artificial Cells, Nanomedicine, and Biotechnology*.

[50] Zhang Y., Bai J., Si W., Yuan S., Li Y., Chen X. (2020). SLC39A7, regulated by miR-139-5p, induces cell proliferation, migration and inhibits apoptosis in gastric cancer via Akt/mTOR signaling pathway. *Bioscience Reports*.

[51] Wei Y., Dong J., Li F., Wei Z., Tian Y. (2017). Knockdown of SLC39A7 suppresses cell proliferation, migration and invasion in cervical cancer. *EXCLI journal*.

[52] Sheng N., Yan L., You W. (2017). Knockdown of SLC39A7 inhibits cell growth and induces apoptosis in human colorectal cancer cells. *Acta Biochimica et Biophysica Sinica*.

[53] Cui Y., Yang Y., Ren L. (2019). miR-15a-3p suppresses prostate cancer cell proliferation and invasion by targeting SLC39A7 via downregulating wnt/*β*-catenin signaling pathway. *Cancer Biotherapy and Radiopharmaceuticals*.

