# Supplementary material for: Comprehensive Analysis of Histone Modifications in Hepatocellular Carcinoma Reveals Different Subtypes and Key Prognostic Models
Source: J Oncol. 2022 Aug 1;2022:5961603. doi: 10.1155/2022/5961603 (PMC9359864; doi:10.1155/2022/5961603)
Supplement: Supplementary Materials — Figure S1. Wilcoxon test was used to analyze the differential expression of 66 of 88 epi-PCG between normal tissues and HCC tissues. Figure S2. The relative changes of CDF and area under CDF curve under different k values of the two external data sets HCCDB18(A) and GSE14520(B) and consensus matrix when k = 3. Figure S3. GO and KEGG analysis of differences epi-PCGs between C1 and C2. A : Significantly downregulated epi-PCGs enriched GO terms and KEGG pathway in C1 compared with C2. B : Significantly upregulated epi-PCGs enriched GO terms and KEGG pathway in C1 compared with C2. Figure S4. GO and KEGG analysis of differences epi-PCGs between C1 and C3. A : Significantly downregulated epi-PCGs enriched GO terms and KEGG pathway in C1 compared with C3. B : Significantly up-regulated epi-PCGs enriched GO terms and KEGG pathway in C1 compared with C3. Figure S5. GO and KEGG analysis of differences epi-PCGs between C2 and C3. A : Significantly downregulated epi-PCGs enriched GO terms and KEGG pathway in C2 compared with C3. B : Significantly upregulated epi-PCGs enriched GO terms and KEGG pathway in C2 compared with C3. Figure S6. A : Correlation between methylation of GMPS promoter region and gene expression. B : Correlation between methylation of SLC39A7promoter region and gene expression. C : Correlation between methylation of SPP1 promoter region and gene expression. D : Correlation between methylation of UCK2 promoter region and gene expression. E : Distribution difference of four gene expression in chemotherapy response group. F : Distribution difference of four gene expression in radiotherapy response group. Table S1. Clinicopathological features between the training set and the validation set. [file 5961603.f1.zip › 5961603.f1/Table S1 (1).docx]

**Table S1 Chi-square test was used to compare clinicopathological features between the training set and the validation set.**

| **Clinical Features** | **TCGA-Train** | **TCGA-test** | **P-Value** |
| --- | --- | --- | --- |
| **OS** |  |  |  |
| 0 | 122 | 113 | 0.3448 |
| 1 | 60 | 70 |  |
| **T Stage** |  |  |  |
| T1 | 93 | 87 | 0.7379 |
| T2 | 47 | 44 |  |
| T3 | 34 | 44 |  |
| T4 | 7 | 6 |  |
| TX | 1 | 2 |  |
| **N Stage** |  |  |  |
| N0 | 126 | 122 | 0.5651 |
| N1 | 1 | 3 |  |
| NX | 55 | 58 |  |
| **M Stage** |  |  |  |
| M0 | 135 | 128 | 0.6029 |
| M1 | 1 | 2 |  |
| MX | 46 | 53 |  |
| **Stage** |  |  |  |
| Ⅰ | 92 | 78 | 0.057 |
| Ⅱ | 45 | 39 |  |
| III | 38 | 45 |  |
| Ⅳ | 1 | 3 |  |
| X | 6 | 18 |  |
| **Grade** |  |  |  |
| G1 | 24 | 31 | 0.5575 |
| G2 | 91 | 84 |  |
| G3 | 60 | 58 |  |
| G4 | 6 | 6 |  |
| GX | 1 | 4 |  |
| **Gender** |  |  |  |
| Male | 125 | 121 | 0.6816 |
| Female | 57 | 62 |  |
| **Age** |  |  |  |
| ≤ 60 | 92 | 81 | 0.2722 |
| ＞60 | 90 | 102 |  |
| **Recurrence** |  |  |  |
| YES | 77 | 90 | 0.2252 |
| NO | 105 | 93 |  |
