# Supplementary figures and images for: Comprehensive Analysis of Histone Modifications in Hepatocellular Carcinoma Reveals Different Subtypes and Key Prognostic Models
Source: J Oncol. 2022 Aug 1;2022:5961603. doi: 10.1155/2022/5961603 (PMC9359864; doi:10.1155/2022/5961603)

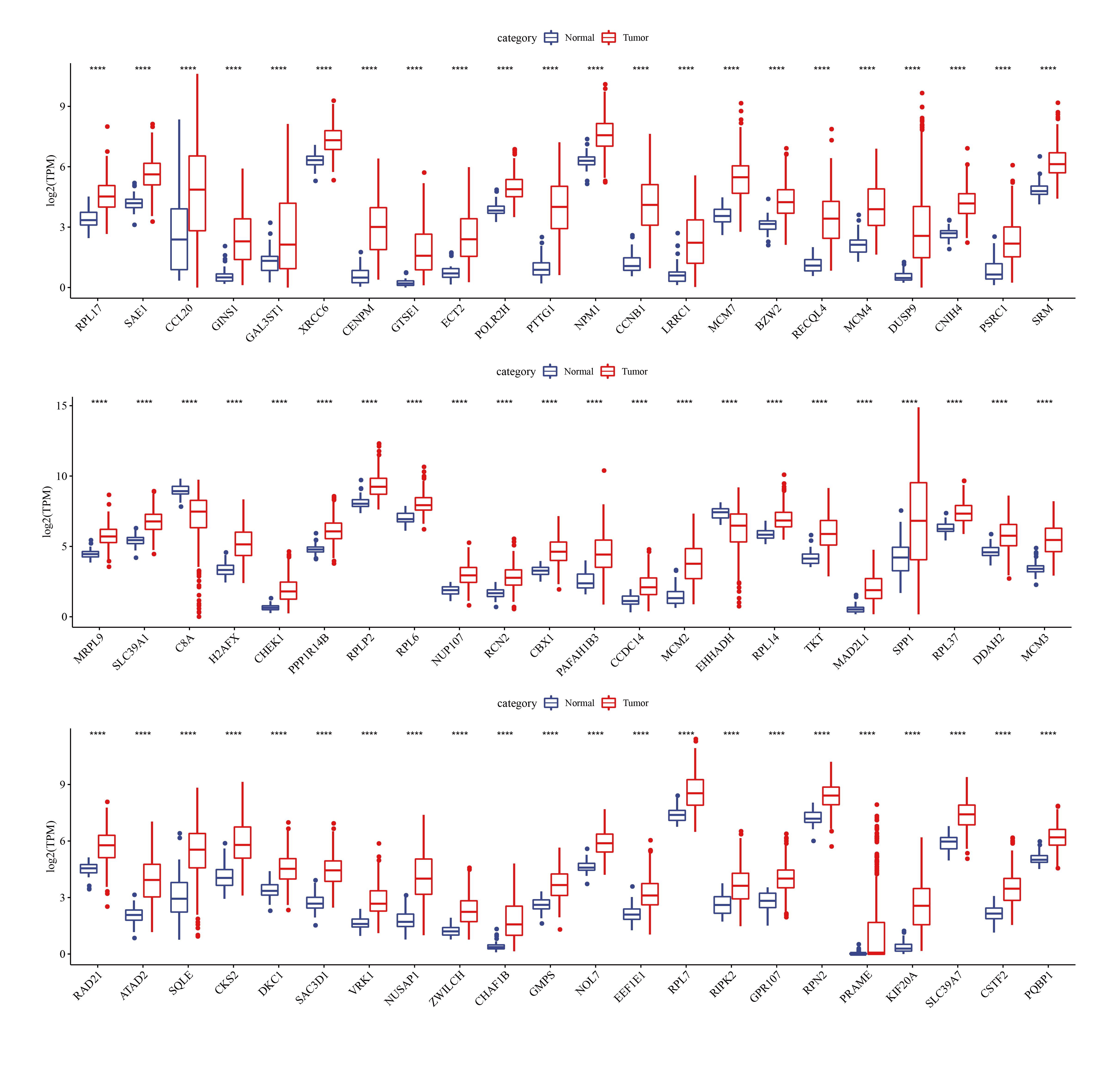

Supplement: Supplementary Materials — Figure S1. Wilcoxon test was used to analyze the differential expression of 66 of 88 epi-PCG between normal tissues and HCC tissues. Figure S2. The relative changes of CDF and area under CDF curve under different k values of the two external data sets HCCDB18(A) and GSE14520(B) and consensus matrix when k = 3. Figure S3. GO and KEGG analysis of differences epi-PCGs between C1 and C2. A : Significantly downregulated epi-PCGs enriched GO terms and KEGG pathway in C1 compared with C2. B : Significantly upregulated epi-PCGs enriched GO terms and KEGG pathway in C1 compared with C2. Figure S4. GO and KEGG analysis of differences epi-PCGs between C1 and C3. A : Significantly downregulated epi-PCGs enriched GO terms and KEGG pathway in C1 compared with C3. B : Significantly up-regulated epi-PCGs enriched GO terms and KEGG pathway in C1 compared with C3. Figure S5. GO and KEGG analysis of differences epi-PCGs between C2 and C3. A : Significantly downregulated epi-PCGs enriched GO terms and KEGG pathway in C2 compared with C3. B : Significantly upregulated epi-PCGs enriched GO terms and KEGG pathway in C2 compared with C3. Figure S6. A : Correlation between methylation of GMPS promoter region and gene expression. B : Correlation between methylation of SLC39A7promoter region and gene expression. C : Correlation between methylation of SPP1 promoter region and gene expression. D : Correlation between methylation of UCK2 promoter region and gene expression. E : Distribution difference of four gene expression in chemotherapy response group. F : Distribution difference of four gene expression in radiotherapy response group. Table S1. Clinicopathological features between the training set and the validation set. [file 5961603.f1.zip › 5961603.f1/Figure S1 (1).jpg]

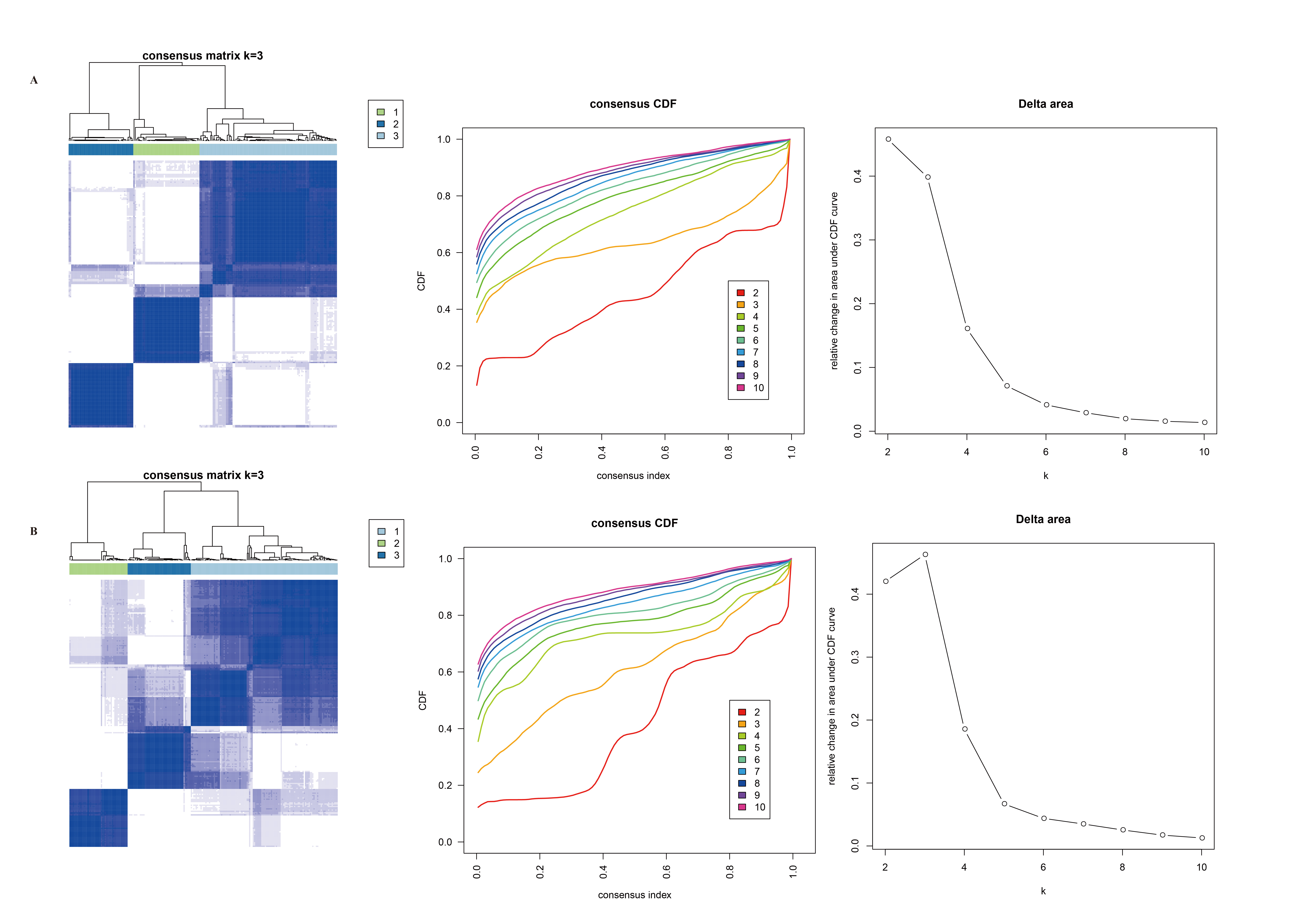

Supplement: Supplementary Materials — Figure S1. Wilcoxon test was used to analyze the differential expression of 66 of 88 epi-PCG between normal tissues and HCC tissues. Figure S2. The relative changes of CDF and area under CDF curve under different k values of the two external data sets HCCDB18(A) and GSE14520(B) and consensus matrix when k = 3. Figure S3. GO and KEGG analysis of differences epi-PCGs between C1 and C2. A : Significantly downregulated epi-PCGs enriched GO terms and KEGG pathway in C1 compared with C2. B : Significantly upregulated epi-PCGs enriched GO terms and KEGG pathway in C1 compared with C2. Figure S4. GO and KEGG analysis of differences epi-PCGs between C1 and C3. A : Significantly downregulated epi-PCGs enriched GO terms and KEGG pathway in C1 compared with C3. B : Significantly up-regulated epi-PCGs enriched GO terms and KEGG pathway in C1 compared with C3. Figure S5. GO and KEGG analysis of differences epi-PCGs between C2 and C3. A : Significantly downregulated epi-PCGs enriched GO terms and KEGG pathway in C2 compared with C3. B : Significantly upregulated epi-PCGs enriched GO terms and KEGG pathway in C2 compared with C3. Figure S6. A : Correlation between methylation of GMPS promoter region and gene expression. B : Correlation between methylation of SLC39A7promoter region and gene expression. C : Correlation between methylation of SPP1 promoter region and gene expression. D : Correlation between methylation of UCK2 promoter region and gene expression. E : Distribution difference of four gene expression in chemotherapy response group. F : Distribution difference of four gene expression in radiotherapy response group. Table S1. Clinicopathological features between the training set and the validation set. [file 5961603.f1.zip › 5961603.f1/Figure S2 (1).jpg]

A

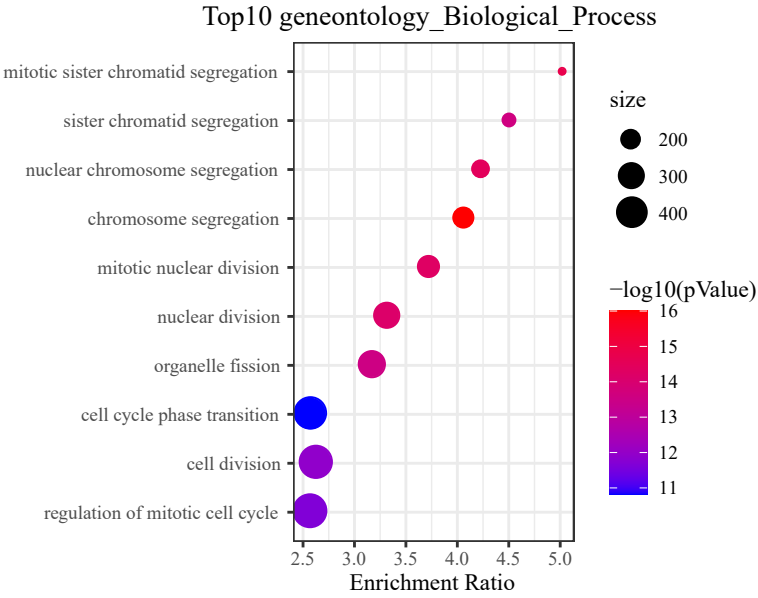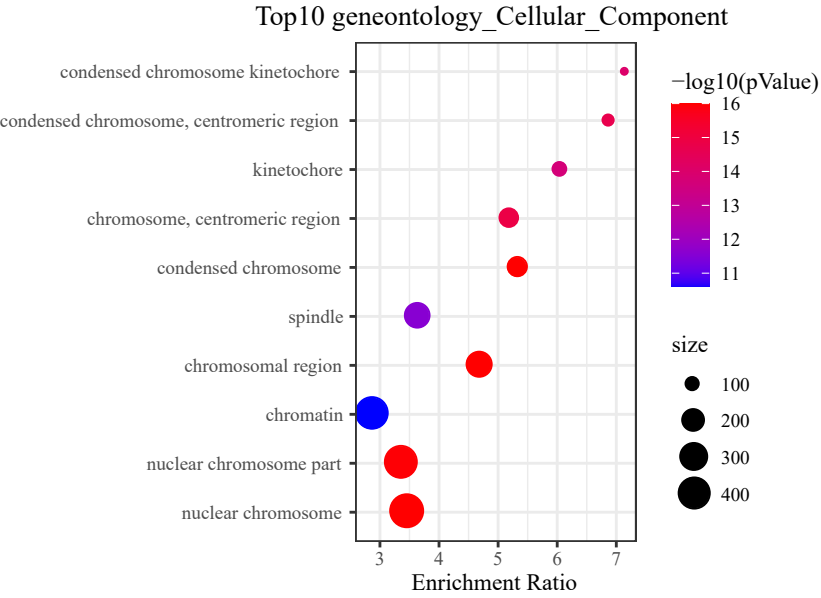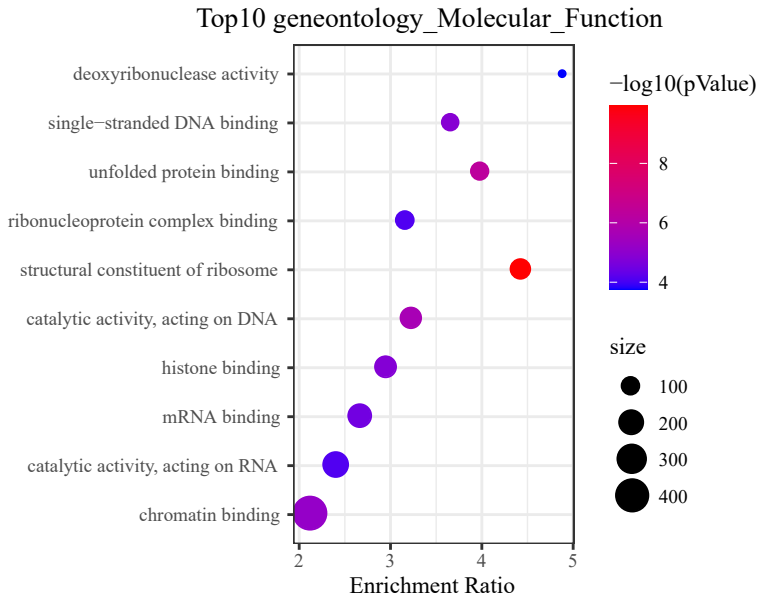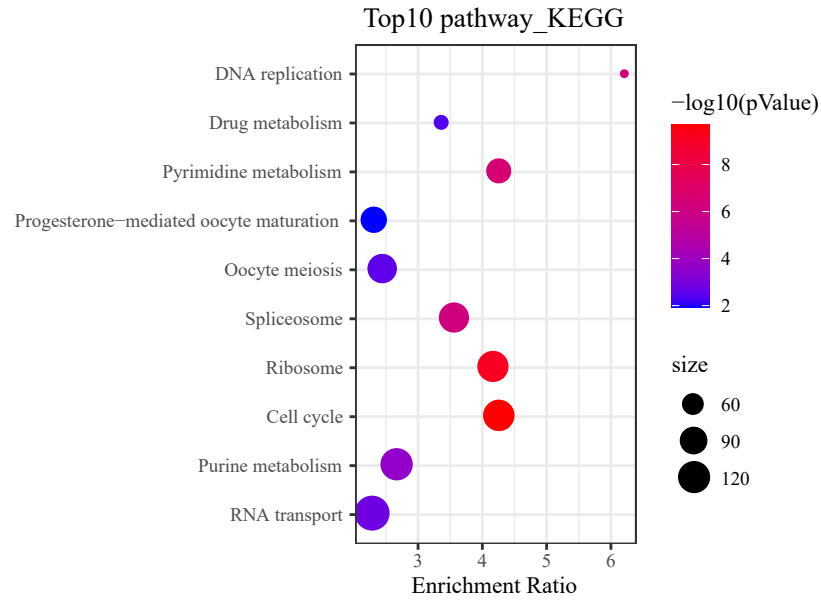

B

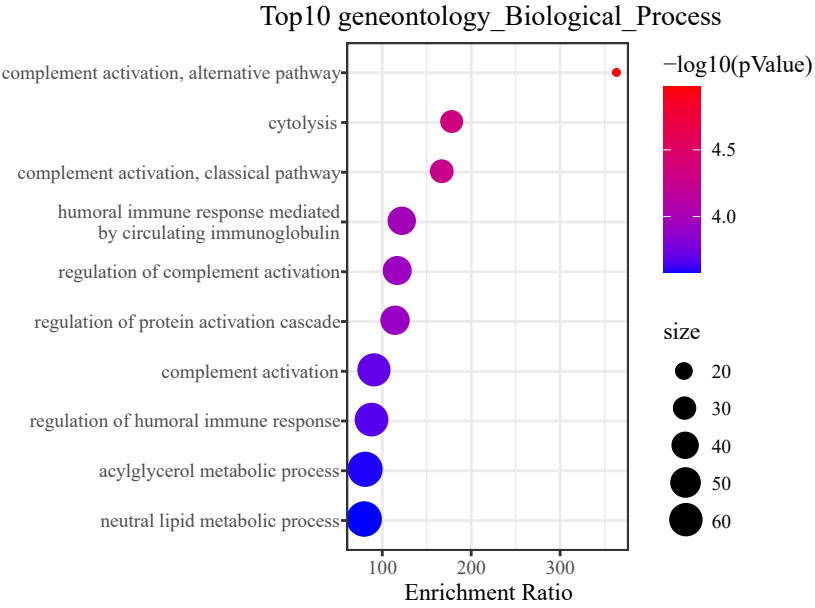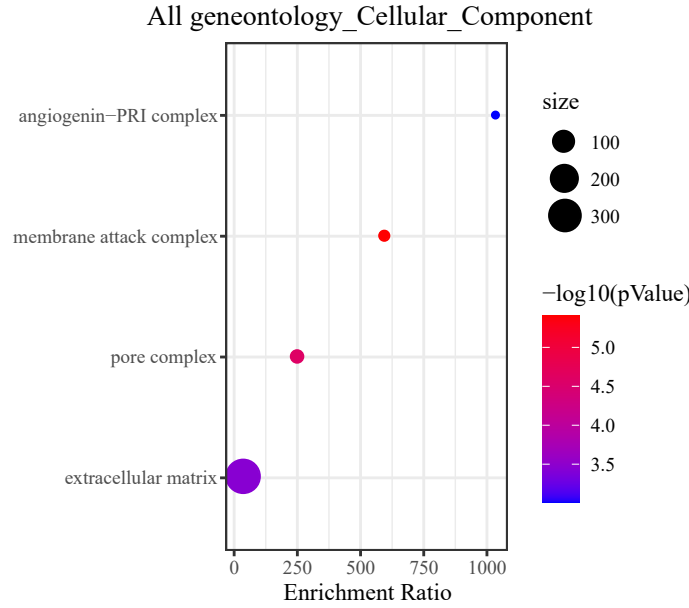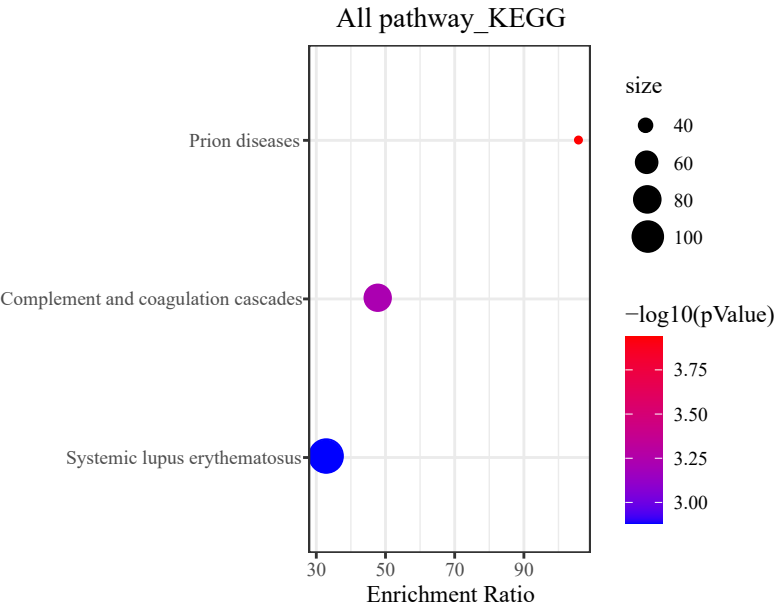

Supplement: Supplementary Materials — Figure S1. Wilcoxon test was used to analyze the differential expression of 66 of 88 epi-PCG between normal tissues and HCC tissues. Figure S2. The relative changes of CDF and area under CDF curve under different k values of the two external data sets HCCDB18(A) and GSE14520(B) and consensus matrix when k = 3. Figure S3. GO and KEGG analysis of differences epi-PCGs between C1 and C2. A : Significantly downregulated epi-PCGs enriched GO terms and KEGG pathway in C1 compared with C2. B : Significantly upregulated epi-PCGs enriched GO terms and KEGG pathway in C1 compared with C2. Figure S4. GO and KEGG analysis of differences epi-PCGs between C1 and C3. A : Significantly downregulated epi-PCGs enriched GO terms and KEGG pathway in C1 compared with C3. B : Significantly up-regulated epi-PCGs enriched GO terms and KEGG pathway in C1 compared with C3. Figure S5. GO and KEGG analysis of differences epi-PCGs between C2 and C3. A : Significantly downregulated epi-PCGs enriched GO terms and KEGG pathway in C2 compared with C3. B : Significantly upregulated epi-PCGs enriched GO terms and KEGG pathway in C2 compared with C3. Figure S6. A : Correlation between methylation of GMPS promoter region and gene expression. B : Correlation between methylation of SLC39A7promoter region and gene expression. C : Correlation between methylation of SPP1 promoter region and gene expression. D : Correlation between methylation of UCK2 promoter region and gene expression. E : Distribution difference of four gene expression in chemotherapy response group. F : Distribution difference of four gene expression in radiotherapy response group. Table S1. Clinicopathological features between the training set and the validation set. [file 5961603.f1.zip › 5961603.f1/Figure S3-modified.pdf]

A

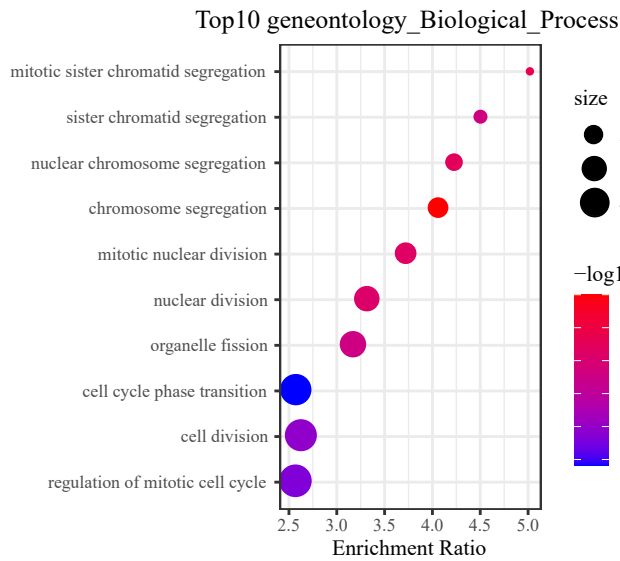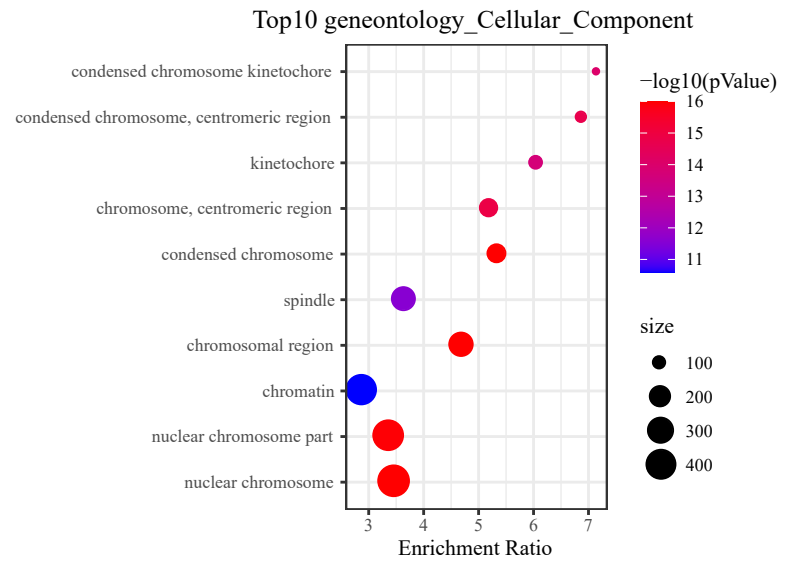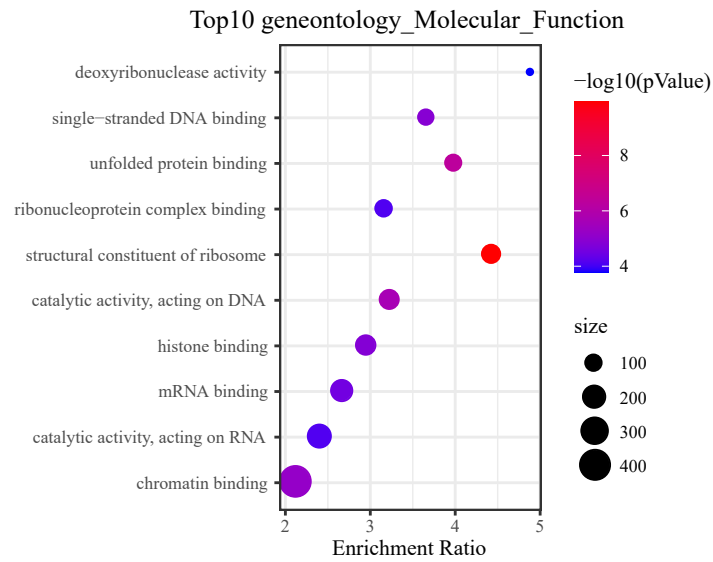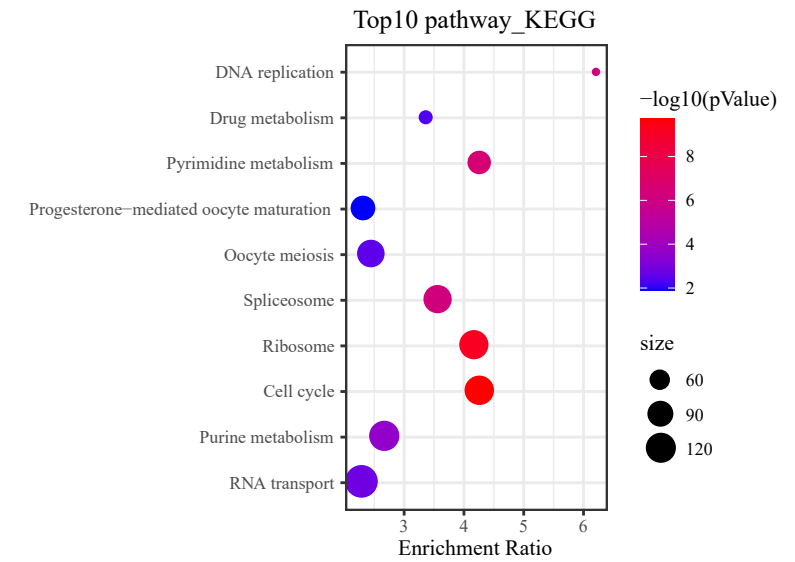

B

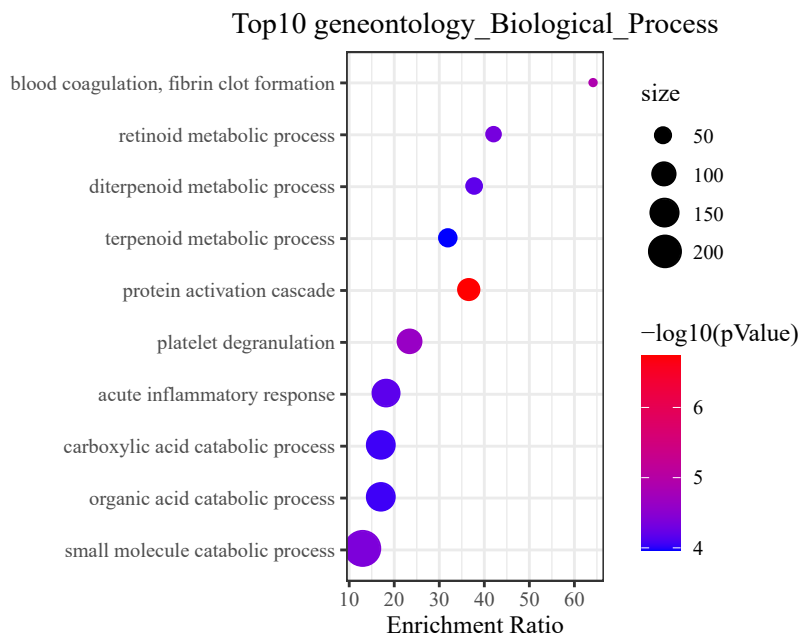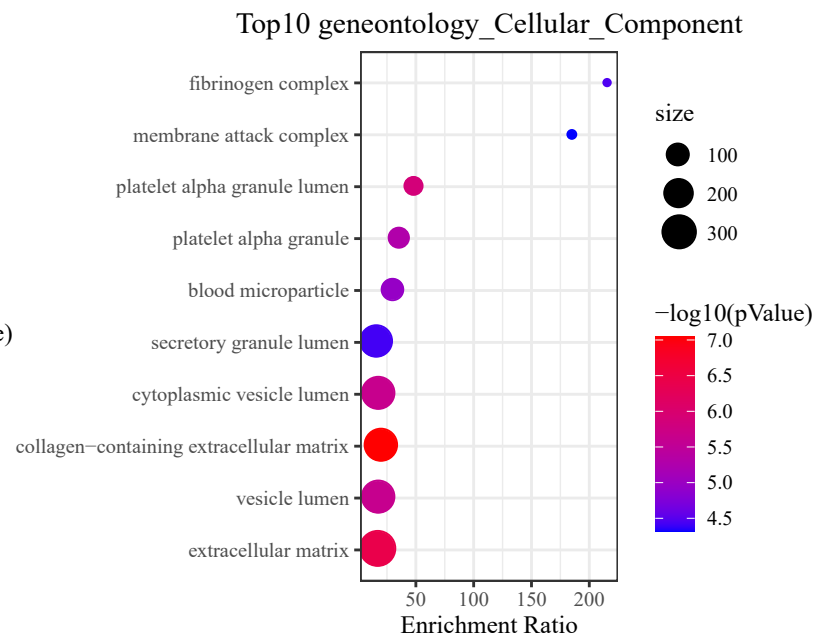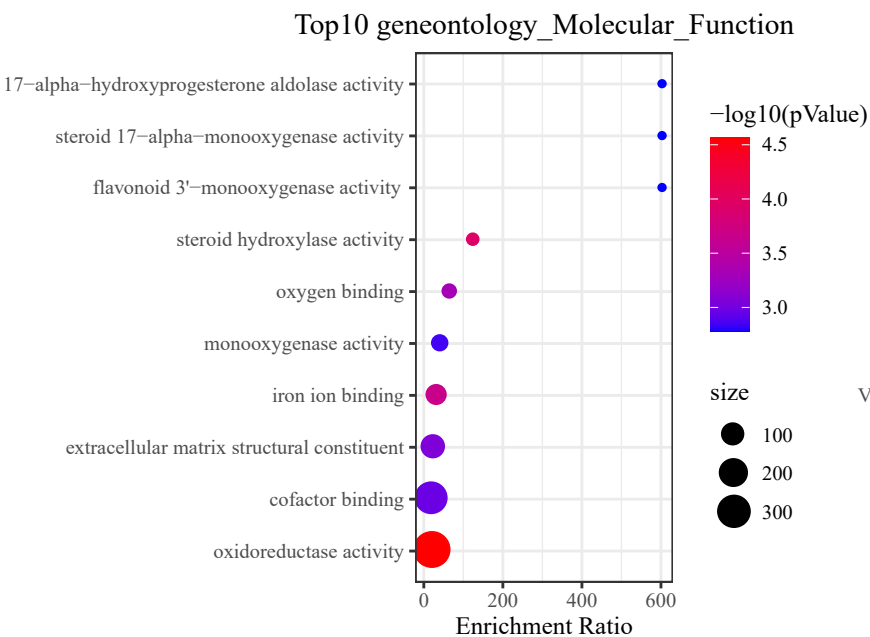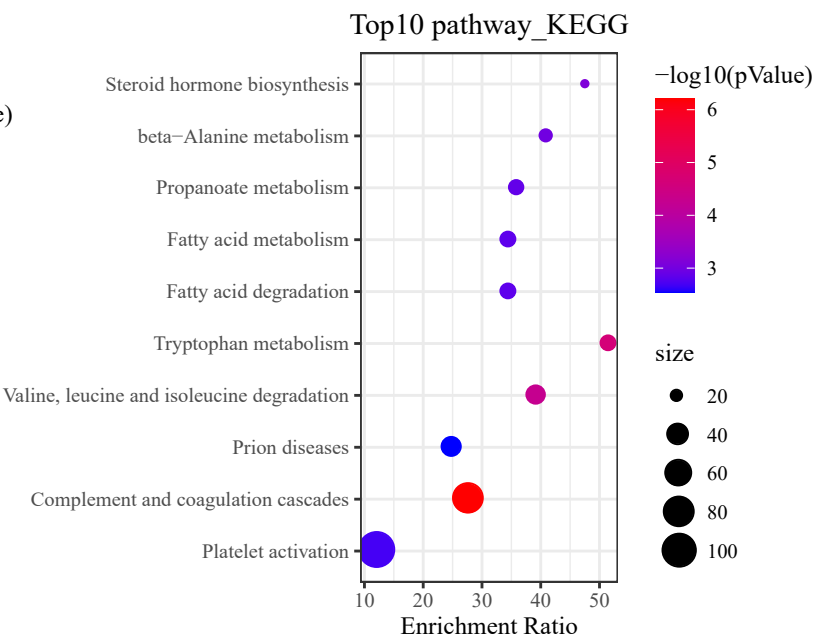

Supplement: Supplementary Materials — Figure S1. Wilcoxon test was used to analyze the differential expression of 66 of 88 epi-PCG between normal tissues and HCC tissues. Figure S2. The relative changes of CDF and area under CDF curve under different k values of the two external data sets HCCDB18(A) and GSE14520(B) and consensus matrix when k = 3. Figure S3. GO and KEGG analysis of differences epi-PCGs between C1 and C2. A : Significantly downregulated epi-PCGs enriched GO terms and KEGG pathway in C1 compared with C2. B : Significantly upregulated epi-PCGs enriched GO terms and KEGG pathway in C1 compared with C2. Figure S4. GO and KEGG analysis of differences epi-PCGs between C1 and C3. A : Significantly downregulated epi-PCGs enriched GO terms and KEGG pathway in C1 compared with C3. B : Significantly up-regulated epi-PCGs enriched GO terms and KEGG pathway in C1 compared with C3. Figure S5. GO and KEGG analysis of differences epi-PCGs between C2 and C3. A : Significantly downregulated epi-PCGs enriched GO terms and KEGG pathway in C2 compared with C3. B : Significantly upregulated epi-PCGs enriched GO terms and KEGG pathway in C2 compared with C3. Figure S6. A : Correlation between methylation of GMPS promoter region and gene expression. B : Correlation between methylation of SLC39A7promoter region and gene expression. C : Correlation between methylation of SPP1 promoter region and gene expression. D : Correlation between methylation of UCK2 promoter region and gene expression. E : Distribution difference of four gene expression in chemotherapy response group. F : Distribution difference of four gene expression in radiotherapy response group. Table S1. Clinicopathological features between the training set and the validation set. [file 5961603.f1.zip › 5961603.f1/Figure S4-modified.pdf]

A

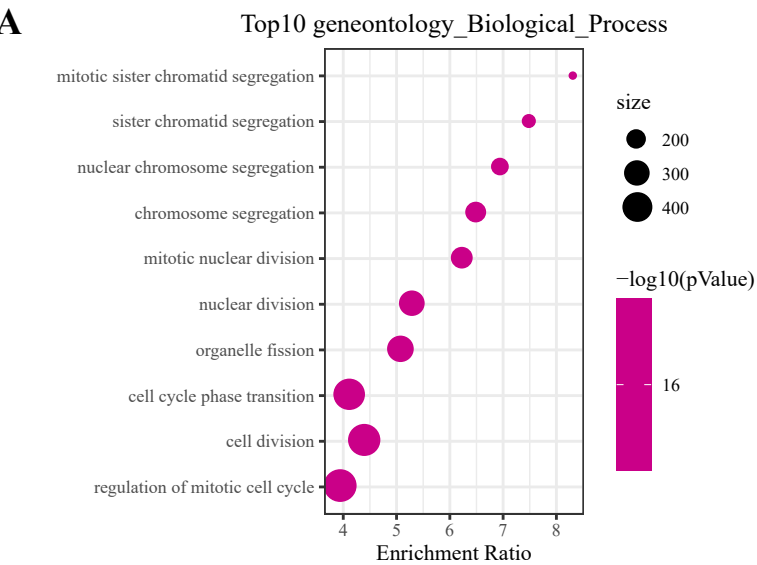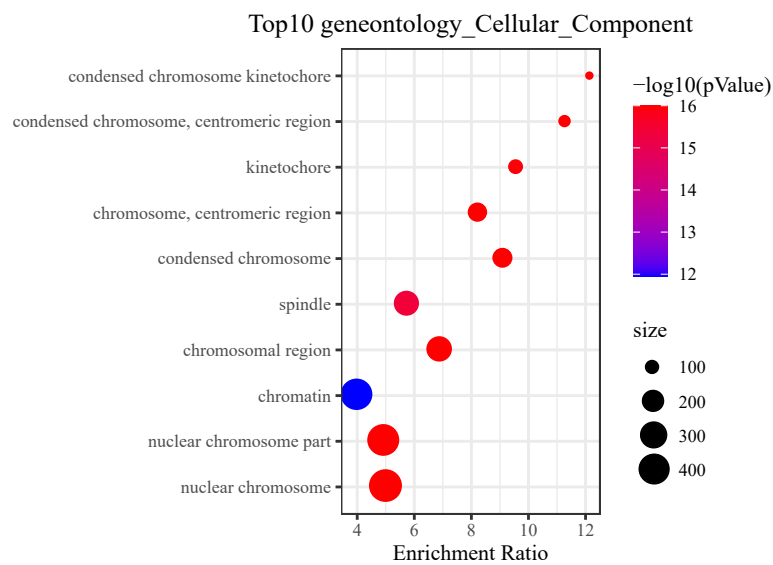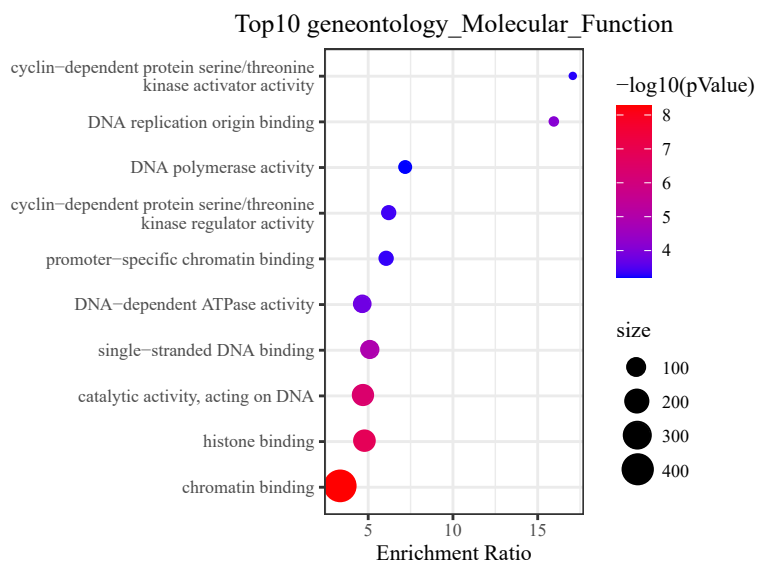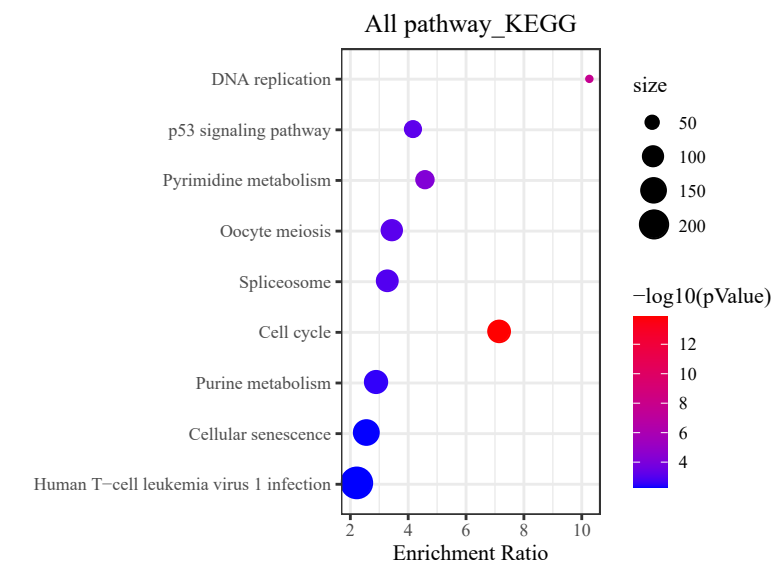

B

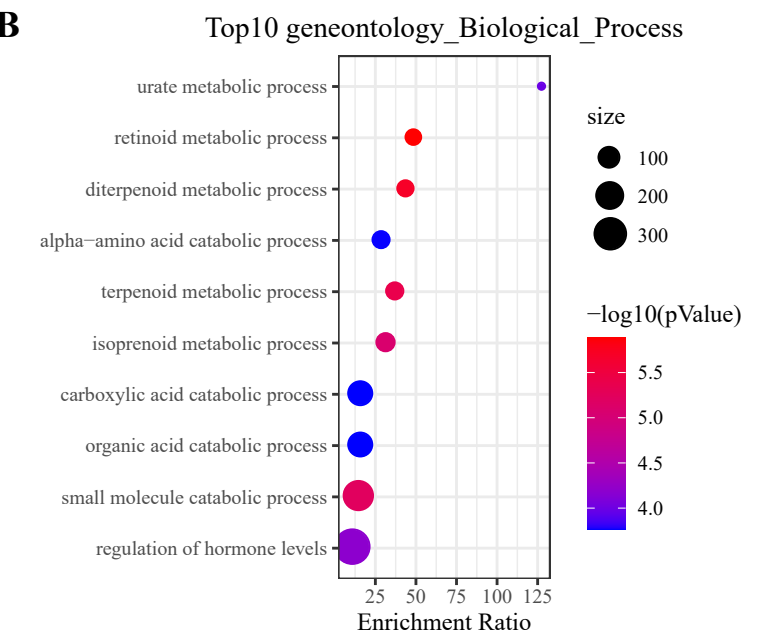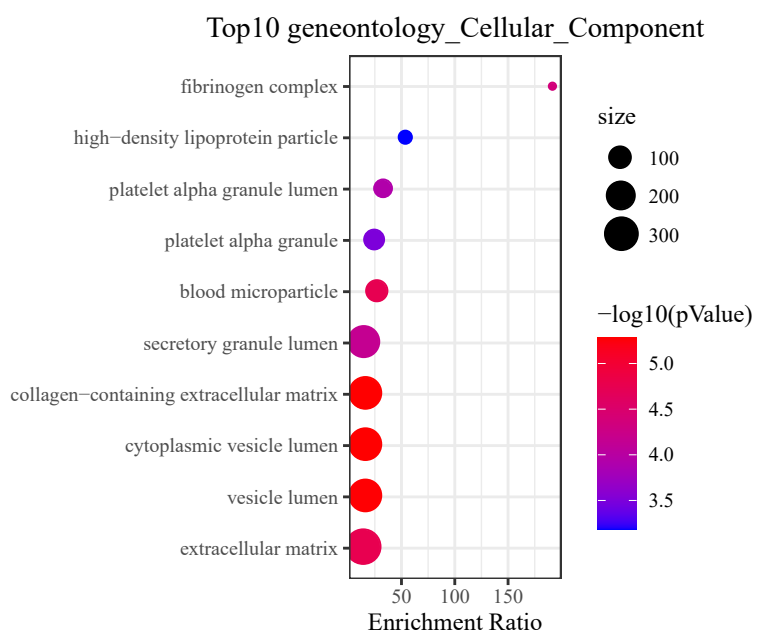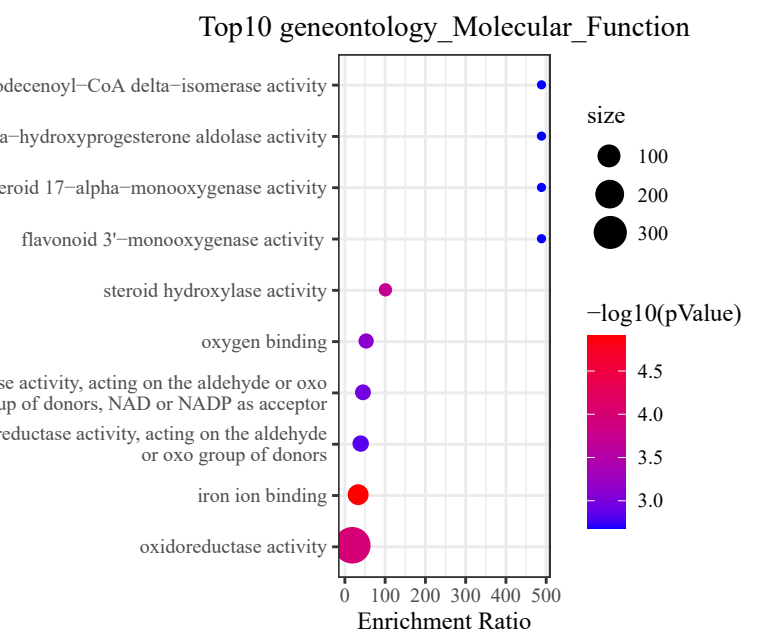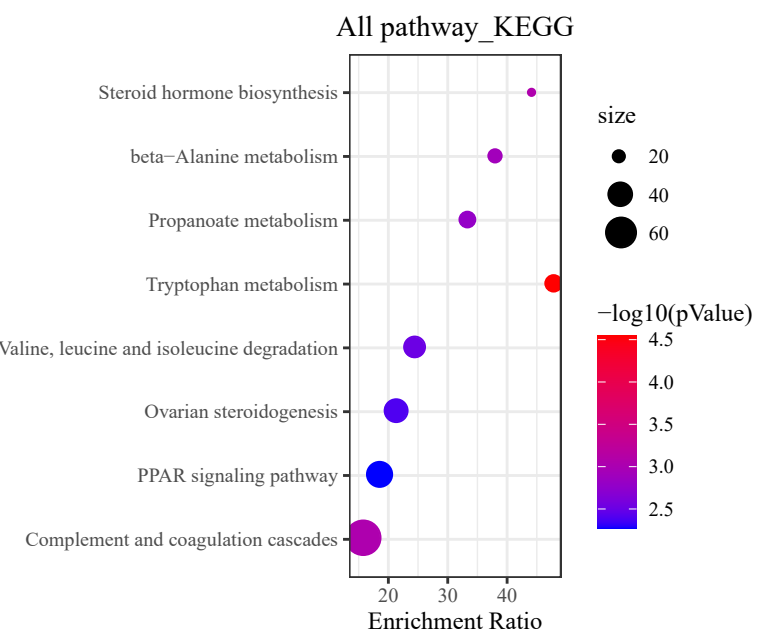

Supplement: Supplementary Materials — Figure S1. Wilcoxon test was used to analyze the differential expression of 66 of 88 epi-PCG between normal tissues and HCC tissues. Figure S2. The relative changes of CDF and area under CDF curve under different k values of the two external data sets HCCDB18(A) and GSE14520(B) and consensus matrix when k = 3. Figure S3. GO and KEGG analysis of differences epi-PCGs between C1 and C2. A : Significantly downregulated epi-PCGs enriched GO terms and KEGG pathway in C1 compared with C2. B : Significantly upregulated epi-PCGs enriched GO terms and KEGG pathway in C1 compared with C2. Figure S4. GO and KEGG analysis of differences epi-PCGs between C1 and C3. A : Significantly downregulated epi-PCGs enriched GO terms and KEGG pathway in C1 compared with C3. B : Significantly up-regulated epi-PCGs enriched GO terms and KEGG pathway in C1 compared with C3. Figure S5. GO and KEGG analysis of differences epi-PCGs between C2 and C3. A : Significantly downregulated epi-PCGs enriched GO terms and KEGG pathway in C2 compared with C3. B : Significantly upregulated epi-PCGs enriched GO terms and KEGG pathway in C2 compared with C3. Figure S6. A : Correlation between methylation of GMPS promoter region and gene expression. B : Correlation between methylation of SLC39A7promoter region and gene expression. C : Correlation between methylation of SPP1 promoter region and gene expression. D : Correlation between methylation of UCK2 promoter region and gene expression. E : Distribution difference of four gene expression in chemotherapy response group. F : Distribution difference of four gene expression in radiotherapy response group. Table S1. Clinicopathological features between the training set and the validation set. [file 5961603.f1.zip › 5961603.f1/Figure S5-modified.pdf]

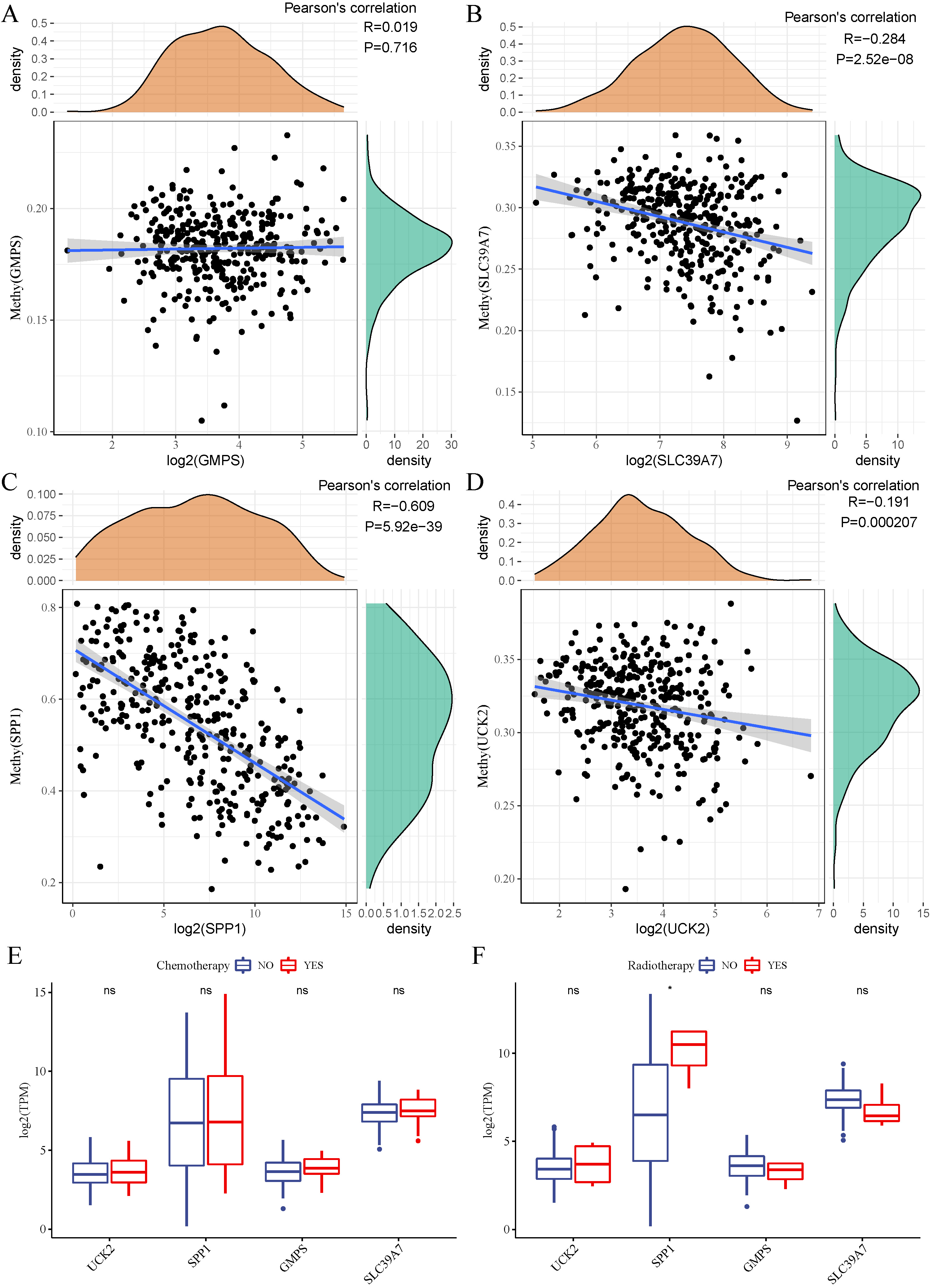

Supplement: Supplementary Materials — Figure S1. Wilcoxon test was used to analyze the differential expression of 66 of 88 epi-PCG between normal tissues and HCC tissues. Figure S2. The relative changes of CDF and area under CDF curve under different k values of the two external data sets HCCDB18(A) and GSE14520(B) and consensus matrix when k = 3. Figure S3. GO and KEGG analysis of differences epi-PCGs between C1 and C2. A : Significantly downregulated epi-PCGs enriched GO terms and KEGG pathway in C1 compared with C2. B : Significantly upregulated epi-PCGs enriched GO terms and KEGG pathway in C1 compared with C2. Figure S4. GO and KEGG analysis of differences epi-PCGs between C1 and C3. A : Significantly downregulated epi-PCGs enriched GO terms and KEGG pathway in C1 compared with C3. B : Significantly up-regulated epi-PCGs enriched GO terms and KEGG pathway in C1 compared with C3. Figure S5. GO and KEGG analysis of differences epi-PCGs between C2 and C3. A : Significantly downregulated epi-PCGs enriched GO terms and KEGG pathway in C2 compared with C3. B : Significantly upregulated epi-PCGs enriched GO terms and KEGG pathway in C2 compared with C3. Figure S6. A : Correlation between methylation of GMPS promoter region and gene expression. B : Correlation between methylation of SLC39A7promoter region and gene expression. C : Correlation between methylation of SPP1 promoter region and gene expression. D : Correlation between methylation of UCK2 promoter region and gene expression. E : Distribution difference of four gene expression in chemotherapy response group. F : Distribution difference of four gene expression in radiotherapy response group. Table S1. Clinicopathological features between the training set and the validation set. [file 5961603.f1.zip › 5961603.f1/Figure S6-modiifed.jpg]
